# Synthesis, Physicochemical Characterization, In Vitro 2D/3D Human Cell Culture, and In Vitro Aerosol Dispersion Performance of Advanced Spray Dried and Co-Spray Dried Angiotensin (1—7) Peptide and PNA5 with Trehalose as Microparticles/Nanoparticles for Targeted Respiratory Delivery as Dry Powder Inhalers

**DOI:** 10.3390/pharmaceutics13081278

**Published:** 2021-08-17

**Authors:** Wafaa Alabsi, Maria F. Acosta, Fahad A. Al-Obeidi, Meredith Hay, Robin Polt, Heidi M. Mansour

**Affiliations:** 1 Department of Chemistry & Biochemistry, The University of Arizona, Tucson, AZ 85721, USA; alabsi@pharmacy.arizona.edu (W.A.); fahada2@email.arizona.edu (F.A.A.-O.); polt@u.arizona.edu (R.P.); 2 Skaggs Pharmaceutical Sciences Center, College of Pharmacy, The University of Arizona, Tucson, AZ 85721, USA; acosta@pharmacy.arizona.edu; 3 The BIO5 Institute, The University of Arizona, Tucson, AZ 85721, USA; mhay@arizona.edu; 4 Department of Physiology, The University of Arizona, Tucson, AZ 85721, USA; 5 Evelyn F. McKnight Brain Institute, The University of Arizona, Tucson, AZ 85721, USA; 6 Division of Translational & Regenerative Medicine, College of Medicine, The University of Arizona, Tucson, AZ 85721, USA

**Keywords:** solid phase peptide synthesis, SPPS, angiotensin, glycopeptide, brain, in vitro 2D/3D cell culture, air–liquid interface, ALI, cell viability, transepithelial electrical resistance, TEER, partitioning, solubility

## Abstract

The peptide hormone Angiotensin (1—7), Ang (1—7) or (Asp-Arg-Val-Tyr-Ile-His-Pro), is an essential component of the renin–angiotensin system (RAS) peripherally and is an agonist of the Mas receptor centrally. Activation of this receptor in the CNS stimulates various biological activities that make the Ang (1—7)/MAS axis a novel therapeutic approach for the treatment of many diseases. The related O-linked glycopeptide, Asp-Arg-Val-Tyr-Ile-His-Ser-(O-β-D-Glc)-amide (PNA5), is a biousian revision of the native peptide hormone Ang (1—7) and shows enhanced stability in vivo and greater levels of brain penetration. We have synthesized the native Ang (1—7) peptide and the glycopeptide, PNA5, and have formulated them for targeted respiratory delivery as inhalable dry powders. Solid phase peptide synthesis (SPPS) successfully produced Ang (1—7) and PNA5. Measurements of solubility and lipophilicity of raw Ang (1—7) and raw PNA5 using experimental and computational approaches confirmed that both the peptide and glycopeptide have high-water solubility and are amphipathic. Advanced organic solution spray drying was used to engineer the particles and produce spray-dried powders (SD) of both the peptide and the glycopeptide, as well as co-spray-dried powders (co-SD) with the non-reducing sugar and pharmaceutical excipient, trehalose. The native peptide, glycopeptide, SD, and co-SD powders were comprehensively characterized, and exhibited distinct glass transitions (T_g_) consistent with the amorphous glassy state formation with T_g_s that are compatible with use in vivo. The homogeneous particles displayed small sizes in the nanometer size range and low residual water content in the solid-state. Excellent aerosol dispersion performance with a human DPI device was demonstrated. In vitro human cell viability assays showed that Ang (1—7) and PNA5 are biocompatible and safe for different human respiratory and brain cells.

## 1. Introduction

The renin–angiotensin system (RAS) is a hormone system known for its blood pressure regulation roles, fluid homeostasis, and atherogenesis; thus, it plays important roles in cardiovascular disease [1]. RAS is a complex cascade of peptides and enzymes including Ang I and II, angiotensin-converting enzymes 1 and 2 (ACE1, ACE2), angiotensin A, angiotensin (1—7) (Ang (1—7)), Mas receptor, Mas-related G coupled protein receptor member D (MrgD), and almandine [1,2,3]. The protein angiotensinogen is metabolized by renin to the decapeptide angiotensin I (Ang I) that circulates without interacting with any receptors. Ang I can then be cleaved by ACE to Ang II, the primary active peptide of the renin–angiotensin system. Ang II activates two angiotensin receptors (GPCRs): angiotensin receptor type 1 (AT1) and angiotensin receptor type 2 (AT2), which together regulate blood pressure. The action of Ang II can become pathological, causing vasoconstriction, inflammation, fibrosis, cellular growth/migration, and fluid retention [1,3].

The cleavage of a single amino acid from Ang II by ACE2 produces the heptamer Ang (1—7), which acts via the Mas receptor. It is a potent vasodilator that can counteract the adverse effects of the Ang II/AT1 axis, including inflammation. The stimulation of the ACE2/Ang (1—7)/MAS axis of the RAS represents a novel therapeutic approach for the potential treatment of diverse disease states. Ang (1—7) protects mainly neurons in the CNS, and can also prevent axon demyelination via oligodendrocytes [4]. Ang (1—7) has antioxidative and anti-inflammatory effects thought to attenuate the development of hypertension; slow the pathological progress of atherosclerosis; and prevent thrombogenic events that contribute to a reduced risk of ischemic stroke, reduced cerebral infarct size, and amelioration of neurological deficits [5]. Ang (1—7) has been reported as an antinociceptive agent in cancer-induced bone pain [6]. Ang (1—7) has cardioprotective effects; treatment with Ang (1—7) attenuated Ang II-induced cardiac hypertrophy, attenuated cardiac fibrosis without interfering with blood pressure, and produced vasodilation in aortic rings and several vascular areas [7]. ACE2 appears to be critically involved in pathophysiological processes in the lungs since Ang (1—7) has been reported to reduce lung inflammation, fibrosis, and pulmonary arterial hypertension [2,8,9]. Moreover, ACE2 has recently been identified as the SARS-CoV-2 receptor site responsible for COVID-19. After virus entry, ACE2 levels decrease, lowering Ang (1—7) production from Ang II [10]. ACE2 is highly expressed in the lungs, cardiovascular system, kidneys, gut, central nervous system, and adipose tissue [11]. Although the relationship between the RAS pathway and COVID-19 is not yet completely clear, the ACE2/Ang (1—7) axis can be manipulated to mitigate SARS-induced tissue injuries, representing a potential target for therapeutic intervention [11].

The use of native peptides as therapeutics is challenging due to their inherent instability in vivo, giving rise to short half-lives of the biologically active peptide and low permeability across biological membranes. Thus, several strategies have been successfully employed to improve the native peptides’ stability, circulation time, safety, immune tolerance, efficacy, and biodistribution. Some of these strategies are the use of unnatural amino acids, modifications such as lipidation, PEGylation, conjugation to antibodies, and stapled peptides [12,13,14]. One of the promising strategies is glycosylation, an effective synthetic approach that introduces carbohydrate moieties to peptides [15]. Some advantages of peptide glycosylation include targeting specific organs, enhancing biodistribution in tissues, improving penetration through biological membranes, increasing metabolic stability, lowering the clearance rate, enhancing receptor-binding, protecting amino acids’ side chain from oxidation, and stabilizing the physical properties of peptides, such as precipitation, aggregation and thermal and kinetic denaturation [16,17,18,19]. As Ang (1—7) is a native bioactive peptide with all above-mentioned therapeutic potential, our group developed a derivative of it via the glycosylation strategy. Glycopeptide drugs based on angiotensin hormones benefit from an intrinsic affinity for their target receptors; they show high biological activity and target specificity at their intended receptors; have low toxicity; and produce greatly reduced (liver mediated) drug–drug interactions relative to classical small molecule drugs [12,15,20,21]. Glycosylation of Ang (1—7) (DRVYIHP) replaces the seventh residue (proline) with serine and has a glucoside attached to the serine, and is amidated at the C-terminus resulting in Ang-1-6-Ser-O-Glu-NH_2_ (PNA5) [15,22]. PNA5 shows enhanced physiological activities over the native hormone, such as increased stability, bioavailability, and blood–brain permeability, leading to reduced reactive oxygen species (ROS) formation and decreased systemic and brain inflammation. PNA5 is an excellent candidate and “first-in-class” therapy for the treatment of cognitive impairment and dementia (VCID) and other inflammatory-related brain diseases [22].

There is increasing attention to formulate therapeutic proteins and peptides as Spray-dried inhalable powder formulations [23,24]. In this study, our goal was to formulate Ang (1—7) and PNA5 as dry powders suitable to be delivered to the respiratory tract (upper and lower). The delivery to the upper respiratory tract allows for needle-free brain delivery via the olfactory route that bypasses the blood–brain barrier (BBB). Moreover, these powders can be used for targeted-lung delivery (lower respiratory tract) as the human lung’s surface area is large and has a highly permeable epithelium, making it easily accessed by an inhaled dose [25]. Hence, pulmonary inhalation offers attractive advantages in delivering high drug concentrations directly to the lungs’ disease site, minimizing systemic bioavailability. Furthermore, delivering Ang (1—7) and PNA5 via inhalation to the lungs may compensate for the endogenous Ang (1—7) depletion due to ACE2 losing its function upon binding to SARS-CoV-2, thus represent a potential therapy for COVID-19 complications (e.g., the cytokine storm) [26].

The spray drying approach to particle engineering offers many advantages in producing particles for dry powder inhalers (DPIs) with favorable physicochemical properties tailored for respiratory delivery. Moreover, increasing the stability of various liquid formulations (e.g., aqueous and organic solutions, emulsions, and suspensions) by rendering them into the solid-state [27,28]. Reducing the amount of residual water present in the solid-state particles by organic solution spray drying will improve the physical and chemical stability. Additionally, particle size, size distribution, and particle morphology can be controlled by altering the conditions used for spray drying [29,30].

Ang (1—7) and its derivative PNA5 were synthesized using published synthetic methods for solid-phase peptide synthesis (SPPS) [31]. The solubility and lipophilicity of Ang (1—7) and PNA5 were determined computationally using molecular operating environment (MOE) software, and experimentally using the shake-flask method (SFM). These two compounds were then formulated in their pure state as SD-powders and as co-SD two-component systems using trehalose as an excipient for intranasal and in-depth lung administration DPI. Several analytical methods were applied to characterize each powder before and after formulations comprehensively: scanning electron microscopy (SEM) to determine particle size, size distribution, and morphology; X-ray powder diffraction (XRPD) to check the crystalline nature (presence or absence of long-range molecular order); differential scanning calorimetry (DSC) to identify the amorphous/crystalline form and the phase transitions upon heating; ATR-FTIR and CRM to determine the chemical composition and confirm the homogeneity of the powders.

The advanced design, development, comprehensive physicochemical characterization, and in vitro aerosol dispersion performance deposition patterns as high performing microparticulate/nanoparticulate DPIs are reported for the first time. In addition, for the first time, the effects of Ang (1—7) peptide and PNA5 glycopeptide on human cell viability were analyzed by measuring the cellular response of the human nasal septum (RPMI2650), Normal human astrocytes (NHA) cells, human blood–brain barrier cells (hCMEC/D3), lung airway cells (H441), and a human alveolar basal epithelial cell (A549) after exposure to different concentrations.

## 2. Materials and Methods

### 2.1. Materials

#### 2.1.1. Solid Phase Peptide Synthesis (SPPS) Reagents

The 2-Chlorotrityl Chloride Resin (Cat# 12996, Lot#000954-551155, 100-300 mesh, substitution 1.43 mmol/g), Rink amide MBHA resin (Cat# 03894, Lot# GRMH0807, grain size 100−200 mesh, substitution 0.83 mmol/g), 6-Chloro-1-hydroxybenzotriazole (ClHOBt) (Cat# 16263, purity 98.8%), Fmoc-L-His (Trt)-OH (Cat# 00713, Lot# 000424-181023-3670, purity 100%), Fmoc-L-Ile-OH (Cat# 02425, Lot# 000565-50827806, purity 99.90%, Fmoc-L-Tyr (tBu)-OH (Cat# 00495, Lot# 000424-160824-36901, purity 99.73%), Fmoc-L-Val-OH (Cat# 02470, Lot# 000424-160701-36001, purity 99.53%), Fmoc-L-Arg(Pbf)-OH (Cat# 01964, Lot# 002992-18110901, purity 99.99%) *N*,*N*-Methylmorpholine (MMP) (Cat# 02333, Lot# 000134-33119256, purity 99.84% (GC)), and 1,8-Diazabicyclo [5.4.0]undec-7-ene (DBU)( Cat# 12723, Lot# 000134-859065560, purity 99.7% (GC)) were obtained from Chem-Impex INT’L INC (Wood Dale, IL, USA). Fmoc-Pro-OH (lot# 9951345), Fmoc-Asp (OtBu)-OH (Lot# 9952107), and 2-(1H-benzotriazol-1-yl)-1,1,3,3-tetramethyluronium hexafluorophosphate (HBTU, Lot#9952720), and *N*,*N*-diisopropylcarbodiimide (DIC) (Lot# 9953116, MW 126.2, Density 0.813 g/mL) were obtained from AAPPTec(Louisville, KY, USA). Anisole (Cas# 100-66-3, density 0.993, purity 99%) was obtained from Aldrich Chemical CO.,INC (Milwaukee, WI, USA). *N*,*N*-Diisopropylethylamine (DIPEA) (Lot# WXBC9624V, ReagentPlus^®^, ≥99%) was obtained from Sigma-Aldrich, Co (St. Louis, MO, USA). *N*-methylpyrrolidone (NMP) (Lot#0000242519, Meets ACS, purity 99.0%) was obtained from VWR Chemicals BDH^®^ (Radnor, PA, USA). Dichloromethane (DCM) (Lot# 198043, certified as ACS, purity 99.5%) was obtained from Fisher Chemical (Geel, Belguim). *N*,*N*-Dimethylformamide (DMF) (Cas# 68-12-2, GR ACS, purity ≥99.8%), and Acetic anhydride (Cas# 108-24-7, GR ACS, purity ≥99.0%) were from EMD Millipore Corporation an Affiliate of Merck. (Darmstadt, Germany). Piperidine (Lot# W18A008, purity 99%), and Hydrazine monohydrate (Lot# Q19E033, purity 98 + %) were obtained from Alfa Aesar (Ward Hill, MA, USA). Trifluoroacetic acid (TFA) (Cas#76-05-1, Lot# 001271N21F), and Triethylsilane (TES) (Cas# 617-86-7, Lot# 005243M15F) were obtained from Oakwood chemical (Columbia, South Carolina, USA). Ether Anhydrous (Batch# 0000244492, purity ≥99.0%) was obtained from Avantor–J.T.Baker^®^ (Radnor, PA, USA).

#### 2.1.2. Advanced Spray Drying and Physicochemical Characterization Reagents

**Figure 1:**
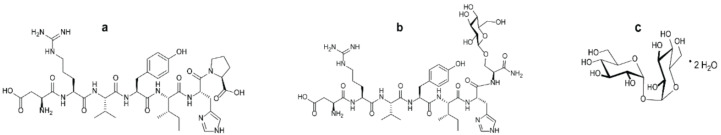
The chemical structures of (**a**) Ang (1—7); (**b**) PNA5; (**c**) Trehalose dihydrate.

Ang (1—7) [C_41_H_62_N_12_O_11_; molecular weight (MW): 898.47 g/mol], shown in (Figure 1a) (ChemDraw Professional 16.0.), was synthesized in Polt’s lab. PNA5 [C_45_H_71_N_13_O_16_; molecular weight (MW): 1049.51 g/mol], shown in (Figure 1b) was synthesized in Polt’s lab. Raw D (+)-Trehalose dihydrate (C_12_H_22_O_11_·2H_2_O; MW: 378.32 g/mol) (Figure 1c) was obtained from Acros Organics (Fair Lawn, NJ, USA). Methanol (HPLC grade, ACS-certified grade, purity 99.9%) was obtained from Fisher Scientific (Fair Lawn, NJ, USA). Ethanol (for HPLC, purity 99.9%) was obtained from Sigma-Aldrich (St. Louis, MO, USA). 1-Octanol (ACS reagent, purity ≥99.9%) was obtained from Sigma-Aldrich, AQUA STAR anhydrous methanol was from EMD Millipore Corporation, an Affiliate of Merck. (Darmstadt, Germany). HYDRANAL^®^-Coulomat AD was obtained from Honeywell Fluka/Sigma-Aldrich Laborchemikalien GmbH (St. Louis, MO, USA). Water was obtained by Milli-Q P-QOD set-up from Millipore (Fair Lawn, NJ, USA) with a resistivity of 18.1 MX cm. All nitrogen gas used for experiments was an ultra-high purity (UHP) nitrogen gas (Cryogenics and gas facility, The University of Arizona, Tucson, AZ, USA). Raw Ang (1—7) and PNA5 were stored in a sealed glass desiccator over Indicating Drierite/Drierite™ desiccant at −80 °C freezer. Raw D (+)-Trehalose dihydrate was stored in a −20 °C freezer. All materials were used as received.

#### 2.1.3. In Vitro Cell Culture Reagents

H441 lung adenocarcinoma cells (ATCC^®^HTB174™, Manassas, VA, USA) and A549 adenocarcinoma human alveolar basal epithelial cells (ATCC^®^CCL-185™, Manassas, VA, USA) were used to represent the lower respiratory tract cell lines, RPMI Medium 1640 (1X) (Gibco, 11875-093, 500 mL, Life Technologies Corporation, Grand Island, NY, USA) were used to grow these two cell lines. RPMI 2650 (ATCC^®^CCL-30™, Manassas, VA, USA) epithelial anaplastic squamous cell carcinoma of the human nasal septum, Eagle’s Minimum Essential Medium (EMEM) (ATCC^®^30-2003™, Manassas, VA, USA) were used. EMEM media was supplemented by 50 mL of Fetal Bovine Serum (FBS) (Gibco, Qualified One Shot™, Life Technologies Corporation, Grand Island, NY, USA), 5 mL of Pen-Strep (10,000 Unite mL^−1^ penicillin, 10,000 μg/mL streptomycin), and 1 mL of fungizone (250 μg/mL amphotericin).

Cryopreserved cells (single donor) CC-2565 Normal Human Astrocytes (NHA) ≥1,000,000 cells (LonzaLonza Walkersville, Inc. (Walkersville, MD, USA), AGM™ BulletKit™ Kit (CC-3186, Lonza), which contains ABM^™^ (CC-3187, Lonza) Astrocyte basal medium (no growth factors) (500 mL) and AGM™ SingleQuots™ (CC-4123, Lonza) supplements for a complete growth medium, trypsin/EDTA (CC-5012, Lonza) were obtained from Lonza Walkersville, Inc. (Walkersville, MD, USA). Trypsin neutralizing solution (CC-5002, Lonza), and HEPES buffered saline solution (CC-5022, Lonza) were obtained from Lonza Walkersville, Inc. (Walkersville, MD, USA). Blood–Brain Barrier hCMEC/D3 Cell Line Immortalized Cell Line (Cat. # SCC066) Pack size: ≥1 × 10^6^ cells/vial, and EndoGRO™-MV Complete Media Kit (cat. no. SCME004) supplemented with 1 ng/mL FGF-2 (cat. no. GF003) were obtained from Millipore Sigma (Billerica, MA, USA). Collagen Type I, Rat Tail (cat. no. 08-115) (Millipore Sigma (Burlington, MA, USA), and PBS pH7.4 (1X) (Gibco, 10010-023, Life Technologies Limited, Paisley, UK) were used to coat the flasks and plates before start growing or seeding the hCMC/D3 cells.

The 3D human airway epithelia (MucilAir^™^, Epithelix, Geneva, Switzerland) reconstituted in vitro using primary human cells were fully differentiated and functional, under conditions previously described [32,33,34]. The cells were received in 24-well Transwell inserts^®^ in a gel matrix. Once the cells were received, they were transferred into a new 24-well plate with 700 µL of the MucilAir™ media (Epithelix, Geneva, Switzerland) in the basal surface. Media was changed every other day, as described by the vendor [35].

### 2.2. Methods

#### 2.2.1. Solid Phase Peptide Synthesis (SPPS)

The synthesis of Ang (1—7) and its glucoside PNA5 has been previously reported [15,22]. PNA5 glycopeptide, Asp-Arg-Val-Tyr-Ile-His-Ser (O-β-D-Glc) was assembled using standard Fmoc-based solid-phase peptide synthesis on Rink amide-MBHA resin (loading: 0.83 mol/g). The Fmoc-serine glucoside was prepared using published methods [18,31,36,37,38]. The coupling of Fmoc-Ser (OGlc-OAc_6_)-OH (1.3 eq) was done manually using ClHOBt (1.3 eq) and DIC (1.3 eq) in NMP, capping of unused sites on the resin was accomplished with 10% acetic anhydride and 10% DIPEA in DCM. The coupling of the Fmoc–protected amino acids was performed on a Prelude^TM^ automated synthesizer. Coupling was accomplished with 2.5 eq Fmoc-AA and HBTU (3.0 eq) and 12 eq of MMP in DMF.

The Fmoc groups were removed using a mixture of 2% piperidine and 2% DBU in DMF for 10 min with argon bubbling as agitation. The final Fmoc group, as well as the acetyl protecting groups of the sugar moiety, were removed by a 1:1 mixture of hydrazine monohydrate in NMP (H_2_NNH_2_•H_2_O:NMP) with argon agitation for 4 h. The PNA5 was cleaved from the resin with a peptide cleavage cocktail consisting of 89% TFA, 10% DCM, 0.25% TES, 0.25% water, and 0.5% Anisole for 2 h at room temperature, which simultaneously removed the side chain protecting groups.

The synthesis of Ang (1—7), which has the sequence of Asp-Arg-Val-Tyr-Ile-His-Pro-COOH, was identical except using the 2-Cl-trityl resin (loading: 1.43 mol/g) for the C-terminal carboxylate instead of Rink amide-MBHA resin used in PNA5 synthesis. The seventh residue, in this case, was Pro (Fmoc-L-Pro-OH), and no hydrazine monohydrate was required.

For the HPLC purification and characterization of compounds, the crude of Ang (1—7) and PNA5 were precipitated in cold ether, then dissolved in a minimal amount of distilled water, and then lyophilized. The crudes were purified by RP-HPLC Gilson system on a semi-preparative C_18_ Phenomenex column (5 µm, 100 Å, 250 × 21.9 mm) using gradient program mobile phase of (A: 5–80% ACN) vs. (B: 0.1% TFA in H_2_O) over 60 min to give the compounds in a pure form. Then, assessing the purity by analytical HPLC (Inspire C18 5 μm 250 mm × 4.6 mm column) on a Varian LC with a diode array detector system (at 280 nm) employing the same gradient mobile phase program for 15 min. The pure fractions obtained from preparative HPLC purification were frozen at −80 °C and then lyophilized to afford the pure peptides as white and fluffy solids. The same analytical RP-HPLC was used to evaluate the purity of the compounds after lyophilization. The mass measurements of pure synthetic compounds were performed and characterized using mass spectrometry (Bruker AmaZon ion trap mass spectrometer via infusion, Analytical & Biological Mass Spectrometry, Core Facility in BIO5 Keating Bioresearch Building, University of Arizona, Tucson, AZ, USA).

#### 2.2.2. Computational Predictions of Physicochemical Parameters of Ang (1—7) Peptide and PNA5 Glycopeptide

Predicted solubility and lipophilicity of Ang (1—7) and PNA5 was performed using the Molecular Environmental Operations program (MOE) (Molecular Operating Environment (MOE), 2019.01; Chemical Computing Group ULC, 1010 Sherbrooke St. West, Suite #910, Montreal, QC, Canada, H3A 2R7, 2019). Since these physicochemical parameters are crucial in designing new formulations for newly discovered drug candidates, it is beneficial to compare the computational properties to the experimental ones. The main focused descriptors are the ones related to pH titration, protomers population, solubility (h_LogS), and lipophilicity (h_LogP).

#### 2.2.3. Solubility Studies

The solubilities of Ang (1—7) and PNA5 were determined by the SFM using conditions similar to previously reported by our group [24] and in [39,40,41,42]. Each compound’s excess powder was added to a specific volume of different solvents (purified water, phosphate buffered saline (PBS), normal saline (NS), methanol, and ethanol) in closed, light-protected glass vials. The solutions containing excess solid of Ang (1—7) and PNA5 were then capped and agitated at room temperature (25 °C) using a shaker. More powder was added to the vial if the dissolution was complete. The pH value of Ang (1—7) and PNA5 suspensions were adjusted to pH 6.5 (pseudo-isoelectric point calculated by MOE software) to determine the zwitterionic species’ solubilities and ensure the predominant presence of the unionized form of Ang (1—7) and PNA5. The separation of the saturated solution and the precipitate was performed by sedimentation. Aliquots of supernatants were withdrawn from all saturated solutions, diluted with each corresponding solvent, and injected into analytical HPLC. Each compound in each aliquot was measured against a calibration curve using the previously developed analytical HPLC method in our lab. Each compound’s solubility experiments in all solvents were repeated three times (*n* = 3, mean ± standard deviation).

#### 2.2.4. Partitioning and LogP Experiments

The partition coefficient of Ang (1—7) and PNA5 were measured at the room, and physiological temperatures via SFM using conditions similar to previously reported [24,43,44,45]. Each compound’s powder was added into glass vials containing equal volumes of 1-octanol and PBS to achieve a 1 mg/mL concentration. The pH was measured and fixed to 6.5 value to assure the presence of the unionized form of Ang (1—7) and PNA5 mainly. Three vials of each compound were shaken at room temperature, while the other three vials were kept in an incubator at 37 °C for 24 h. After 24 h, the shaking was stopped, and the vials kept equilibrating to ensure complete separation between the 1-octanol and aqueous layers at these specific temperatures. A sample from each layer was withdrawn and analyzed by analytical HPLC to measure the compound’s concentration in each layer then calculate the partition coefficient (P) and logP using the following equations:

(1) $$P\;=\frac{\left[\mathrm{neutral}\;\mathrm{solute}\right]\mathrm{oct}}{\left[\mathrm{neutral}\;\mathrm{solute}\right]\mathrm{Aq}}$$

(2) $$\log P=\log\frac{\left[\mathrm{neutral}\;\mathrm{solute}\right]\mathrm{oct}}{\left[\mathrm{neutral}\;\mathrm{solute}\right]\mathrm{Aq}}$$

#### 2.2.5. HPLC for Solubility and LogP Experiments

The HPLC system is LC-2010HT next-generation HPLC (SHIMADZU Tokyo, Japan), and it is the same as previously used in [24]. This system consists of a degassing unit, low-pressure gradient unit, pump unit, mixer, ultra-fast autosampler, column oven, and a UV-VIS detector with a thermostatted flow cell system. The analysis was performed by a reverse phase HPLC assay, using Phenomenex Prodigy 5 um ODS (3) 250 × 4.6 mm HPLC Column. Ultraviolet detection was done at 280 nm. Mobile phase condition was a gradient of acetonitrile in water with 0.1% trifluoroacetic acid at two different percentages pumped from two pumps. The flow was 1 mL/min, the injection volume was 20 μL; the retention time for Ang (1—7) was ~7.7 min and for PNA5 was ~6.7 min. Quantification for both compounds was determined using peak area and calculated from freshly prepared standard curves.

#### 2.2.6. Preparation of SD and co-SD Particles by Organic Solution Advanced Spray Drying in Closed-Mode

The organic solution advanced spray drying process in the absence of water was performed in closed mode, using a Büchi B-290 Mini Spray Dryer. A high-performance cyclone in closed mode using and connected to the B-295 Inert Loop (Büchi Labortechnik AG, Flawil, Switzerland). The atomizing drying gas was UHP dry nitrogen. Table 1 lists the spray drying conditions for one and two-component powders and the corresponding outlet temperatures for different formulations. The stainless steel two-fluid nozzle tip diameter was 0.7 mm with a 1.5 mm gas cap. All the instrument parameters were maintained constant during all the experiments. The SD and co-SD particles were separated from the nitrogen drying gas in the high-performance cyclone and collected in a sample collector. All SD and co-SD powders were carefully stored in sealed glass vials stored in a sealed glass desiccator, indicating Drierite/Drierite™ desiccant at −80 °C ambient pressure. These are similar conditions, as reported previously by our group [24,28,29,30,46,47,48].

#### 2.2.7. Scanning Electron Microscopy (SEM)

SEM of raw Ang (1—7), raw PNA5, and Trehalose (as supplied by the manufacturer), SD Ang (1—7), SD PNA5, SD Trehalose, co-SD Ang (1—7):Trehalose (25:75) and co-SD PNA5:Trehalose (25:75) powders were evaluated using same conditions reported in [24]. Visual imaging, analysis of particle size, morphology, and surface morphology were achieved by (SEM). Powder samples were attached to aluminum SEM stubs (Ted-Pella, Inc., Redding, CA, USA) using double-sided carbon conductive adhesive Pelco tabs™ (Ted Pella, Inc. Redding, CA, USA). Then, using Anatech Hummer 6.2 (Union City, CA, USA) system, each sample in the stub was sputter-coated with a 7 nm thin film of gold at 20 μA for 90 s under an argon plasma. SEM images were captured at several different magnification levels. SEM images of the powder sample were collected using an FEI Inspect S (FEI, Hillsboro, OR, USA) and using a tungsten source at 30 kV with a working distance of 10 mm–10.4 mm.

#### 2.2.8. Particle Sizing and Size Distribution

The mean size, standard deviation, and size range of each powder particle were determined digitally using SigmaScan Pro5.0.0 (Systat, San Jose, CA, USA). As previously reported [24,28,29,30,46,47,49], representative SEM micrographs for each particle sample at 8000× magnification were analyzed by measuring the diameter of at least 100 particles per powder.

#### 2.2.9. X-ray Powder Diffraction (XRPD)

The presence of long-range molecular order (crystalline) versus the lack of long-range molecular order (amorphous) of Ang (1—7), PNA5, and Trehalose (all unprocessed), SD, and co-SD powders were evaluated using XRPD. The patterns samples patterns were collected at room temperature using conditions previously reported [24]. Thus, each powder sample was loaded on a zero-background single crystal silicon holder. The experiments were performed on a Philips PANalytical X’Pert PRO MPD instrument equipped with the copper X-ray source (Kα radiation with λ = 1.5406 Å) and a high-sensitivity X’celerator X-ray detector between 5.0° and 90.0° (2θ) with a scan rate of 2.00° per min at ambient temperature from a small amount of powder.

#### 2.2.10. Differential Scanning Calorimetry (DSC)

Using conditions similar to previously reported [24,28,29,30,46,47,48,49], thermal analysis and phase transition measurements of Ang (1—7), PNA5, and Trehalose (all unprocessed), SD, and co-SD formulations were studied. The thermograms were obtained using the TA Q1000 differential scanning calorimeter (DSC) (TA Instruments, New Castle, Delaware). A mass of 3–5 mg of each sample was weighed into hermetic anodized aluminum DSC pan. DSC measurements were performed at the heating rates of 5.00 °C/min and 40.0 °C/min from 0.00–300 °C. As a reference, an empty, hermetically sealed aluminum pan was used. The purging gas was nitrogen at a rate of 50 mL/min. Phase transition parameters including the glass transition temperature (T_g_), melting point (T_m_) main phase transition, enthalpy (ΔH), and heat capacity (ΔC_p_) were measured and calculated using TA Universal Analysis (TA Instruments). Moreover, to ensure reproducibility, each experiment was repeated three times.

#### 2.2.11. Hot-Stage Microscopy (HSM) under Cross-Polarizers

The presence or absence of birefringence during the solid-state phase transitions for each powder was observed under a cross-polarizing light HSM, using conditions similar to previously reported [30,46,47,48,49]. HSM experiments were performed using a Leica DMLP cross-polarized microscope (Wetzlar, Germany) coupled with a Mettler FP 80 central processor heating unit and Mettler FP82 hot stage (Columbus, OH, USA). The powder sample was placed on a microscope slide with a cover glass and placed into the hot stage chamber, and heated at a rate of 5.00 °C/min from 25.0–300.0 °C. The images at specific temperatures were digitally captured using a Nikon Coolpix 8800 digital camera (Nikon, Tokyo, Japan) under 10× optical objective and 10× digital zoom.

#### 2.2.12. Karl Fisher Titration (KFT)

All powders’ residual water content was quantified by KFT coulometrically using conditions similar to previously reported [24]. A TitroLine^®^ 7500 trace titrator (SI Analytics, Mainz, Germany) was used. A mass of 1–2 mg of powder sample was dissolved in 0.5 mL AQUA STAR anhydrous methanol and injected the sample solution into a reaction cell filled with Hydranal^®^ Coulomat AD reagent. Then obtain the water content of the sample a simple calculation. All experiments were done in triplicate (*n* = 3).

#### 2.2.13. Confocal Raman Spectroscopy (CRM)

CRM has demonstrated utility in the non-invasive and non-destructive microspectroscopic analysis of DPI formulation using conditions similar to previously reported [24]. Raman spectra were obtained for all powders (as raw and formulated) at 785 nm laser excitation. The Renishaw inVia Reflex (Gloucestershire, UK) was used at the surface, using a 20× magnification objective on a Leica DM2700 optical microscope (Wetzlar, Germany), and equipped with a Renishaw inVia Raman system (Gloucestershire, UK). Measurements were conducted using a 1200 lmm^−1^ grating with a slit width of 65 μm and a thermoelectrically cooled Master Renishaw CCD detector. Raman spectral was performed for each co-SD Ang (1—7):Trehalose (25:75) and co-SD PNA5:Trehalose (25:75) powder at three different spots of the powder. Each point acquired three accumulations using 10 s of detector exposure time per accumulation with spectral scans of 50–4000 cm^−1^ performed on all samples. Spectra were subjected to baseline correction before further analysis using Renishaw WiRE 3.4 software.

#### 2.2.14. Attenuated Total Reflectance (ATR)–Fourier-Transform Infrared (FTIR) Spectroscopy

Conditions similar to those previously reported [24] were used to obtain ATR-FTIR spectra for all powders (processed and unprocessed). A Nicolet IS50R FT-IR spectrometer (Waltham, MA, USA) coupled with an MCT-A detector and a Thunderdome attenuated total reflectance (ATR) (Spectra-Tech, Oak Ridge, TN, USA) accessory with a germanium window was used for all the experiments. Each spectrum was collected for 32 scans at a spectral resolution of 8 cm^−1^ over the wavenumber range of 4000–400 cm^−1^; spectral data were acquired and processed using OMNIC Spectra Software.

#### 2.2.15. In Vitro Aerosol Dispersion Performance

Aerosolization of raw (unprocessed), SD, and co-SD powders was evaluated, following United States Pharmacopeia (USP) chapter <601> specifications on aerosols specifications and using conditions similar to previously reported [24]. The Next Generation Impactor™ (NGI™) (MSP Corporation, Shoreview, Minnesota, USA) was used, coupled with a human DPI device, the Neohaler™ (Novartis, Basel, Switzerland), at an airflow rate (Q) of 60 L/min (adult airflow rate) using a Copley DFM 2000 digital flow meter (Copley Scientific, Nottingham, UK). The mass of deposited powder on each stage was quantified by the gravimetric method using type A/E glass fiber filters with diameter 55 mm (PALL Corporation, Port Washington, New York, NY, USA) and 75 mm (Advantec, Japan) in the NGI gravimetric collection cups (MSP Corporation, Shoreview, MN, USA). All experiments were triplicated (*n* = 3) at ambient temperature and humidity. The emitted dose (ED) was determined as the difference between the initial mass of powder loaded in the capsules and the remaining powder in the Quali-V clear HPMC size 3 inhalation grade capsules (Qualicaps, NC, USA) following aerosolization. The ED (%) Equation (3) was used to express the percentage of ED based on the total dose (TD) used. The fine particle dose (FPD) was defined as the dose deposited on stages 2 to 7. The fine particle fraction (FPF%) Equation (4) was expressed as the ED’s FPD percentage. The respirable fraction (RF%) Equation (5) was used as the percentage of FPD to total deposited dose (DD) on all impactor stages.

(3) $$\mathrm{Emitted}\;\mathrm{Dose}\;\left(\mathrm{ED}\%\right)\;=\frac{\mathrm{ED}}{\mathrm{TD}}\times 100\%$$

(4) $$\mathrm{Fine}\;\mathrm{Particle}\;\mathrm{Fraction}\;\left(\mathrm{FPF}\%\right)\;=\frac{\mathrm{FPD}}{\mathrm{ED}}\times 100\%$$

(5) $$\mathrm{Respirable}\;\mathrm{Fraction}\;\left(\mathrm{RF}\%\right)\;=\frac{\mathrm{FPD}}{\mathrm{DD}}\times 100\%$$

The mass median aerodynamic diameter (MMAD) of aerosol particles and geometric standard deviation (GSD) was calculated using a Mathematica (Wolfram Research Inc., Champaign, IL, USA) program written by Dr. Warren Finlay.

#### 2.2.16. In Vitro 2D Cell Viability of Human Cells

In vitro human cell viability assays were performed to measure the biocompatibility and safety of Ang (1—7) and PNA5 on nasal epithelium RPMI 2650 cells, hCMEC/D3 blood–brain barrier endothelial cells, NHA, A549, and H441 lung cells using resazurin as a marker.

Human nasal epithelial cells (RPMI 2650) with passage numbers between 9 and 13 were used for the experiments. Eagle’s Minimum Essential Medium (EMEM) (ATCC^®^ 30-2003™) supplemented with 10% (*v*/*v*) fetal bovine serum (FBS), 1% (*v*/*v*) Pen-Strep (100 Unit/mL penicillin, 100 μg/mL streptomycin), 0.2% (*v*/*v*) and fungizone (0.5 μg/mL amphotericin) was used to grow cells in a humidified incubator at 37 °C and 5% CO_2_. Normal Human Astrocytes (NHA) at passages 1 and 3 were used for the experiments. A supplements kit for a complete growth medium AGM™ SingleQuots™ (Lonza, CC-4123) was added to the growth medium of Astrocyte basal medium ABM™ (Lonza, CC-3187). Cell culturing and sub-culturing were performed according to the manufacture protocol [50].

The immortalized human cerebral microvascular endothelial (hCMEC/D3) at passages 29 and 31 were seeded on collagen-coated culture flasks. Cells were grown in EndoGRO™-MV Complete Media Kit (cat. no. SCME004) supplemented with one ng/mL FGF-2 (cat. no. GF003) and kept in a humidified incubator at 37 °C and 5% CO_2_. Cell culturing and sub-culturing were performed according to the manufacturing protocol [51].

A549 adenocarcinoma human alveolar basal epithelial cells (ATCC^®^ CCL-185™) and H441 lung adenocarcinoma cells (ATCC^®^HTB174™) were used at passages 9 and 8, respectively, for the experiments. Both cell types were grown in a growth in RPMI Medium 1640 (1X) (Gibco, 11875-093, 500 mL) supplemented with 10% *v*/*v* fetal bovine serum (FBS), 0.2% *v*/*v* fungizone (0.5 μg/mL amphotericin), 1% *v*/*v* Pen-Strep (100 Unit/mL penicillin, 100 μg/mL streptomycin). The cells were kept in a humidified incubator at 37 °C and 5% CO_2_.

For passage, all the cell types were detached when confluence reached about 80% by treating the cells with trypsin-EDTA at 37 °C. The effects of Ang (1—7) and PNA5 on cell proliferation were analyzed by measuring all of the above cell line responses to different concentrations of Ang (1—7) and PNA5 separately. The cells were seeded in 96-well black plates at a specific density and were kept to attach for 48 h. Then, the media containing compounds were removed from each well and replaced by 100 μL of media containing 20 μM resazurin sodium salt (was dissolved in un-supplemental media) and incubated for 4 h at 37 °C and 5% CO_2_. The fluorescence intensity of the metabolite (resorufin) produced by the viable cells was measured at 544 nm (the excitation) and 590 nm (the emission) using the Synergy H1 Multi-Mode Reader (BioTek Instruments Inc., Winooski, VT, USA). The relative cell viability of the cell line was calculated using Equation (6):

(6) $$\mathrm{Relative}\;\mathrm{viability}\;\left(\%\right)=\frac{\mathrm{Sample}\;\mathrm{fluorescence}\;\mathrm{intensity}}{\mathrm{Control}\;\mathrm{fluorescence}\;\mathrm{intensity}}\times 100\%$$

#### 2.2.17. In Vitro 2D Transepithelial Electrical Resistance (TEER) at the Air–Liquid Interface (ALI) of Human Lung Epithelial Cells

Calu-3, a human lung epithelial cell line derived from bronchial submucosal airway region, was used as a model for monolayer integrity in the upper airways, and is known to form tight junctions. Using previously reported similar conditions, cells were grown in a growth medium including Eagle’s minimum essential medium (EMEM), 10% (*v*/*v*) fetal bovine serum (FBS), Pen-Strep (100 U/mL penicillin, 100 µg/mL), Fungizone (0.5 µg/mL amphotericin B, 0.41 µg/mL sodium deoxycholate) in a humidified incubator at 37 °C and 5% CO_2_. After confluence, the cells were seeded at a concentration of 500,000 cells/mL in Costar (Costar 3460, Corning, New York, NY, USA) Trans-well inserts^®^ (0.4 μm polyester membrane, 12 mm for a 12-well plate) from Fisher Scientific (Hampton, NH, USA) with 0.5 mL of media on the apical side and 1.5 mL of media on the basolateral side. Media was changed every other day from the basolateral side. After approximately one week of growth, when the cells looked packed and a complete monolayer was visible under the microscope, TEER values were measured using an EndOhm 12 mm Culture Cup (World Precision Instruments, Sarasota, FL, USA). TEER values of 1000 Ω·cm^2^, were an indicator of a confluent monolayer at liquid-covered culture (LCC). At this point, the media was removed from the apical side in order to facilitate air–liquid interface (ALI) conditions. The TEER responses of the cells were also measured with an EndOhm 12 mm Culture Cup (World Precision Instruments, Sarasota, Florida). For TEER measurements at ALI, 0.5 mL of media was added to the apical side of the each Transwell insert 30 min before the measurement and then immediately removed to return the cells to ALI conditions. After the TEER values reached 500 Ω·cm^2^ (indicating a confluent monolayer at ALI conditions), the cells were exposed to 100 µM of representative SD formulations dissolved in 90:10 media:ethanol to facilitate dissolution. The liquid aerosol formulations were delivered to the Calu-3 cells at ALI using a Penn-Century MicroSprayer^®^ Aerosolizer Model IA-1B (Penn-Century, Inc., Wyndmoor, PA, USA). TEER values were then recorded after 3 h of exposure and then recorded every 24 h up to 7 days after drug exposure using an EndOhm 12 mm Culture Cup (World Precision Instruments, Sarasota, FL, USA), as previously reported [32,33,34,52].

#### 2.2.18. In Vitro Cell Viability of 3D Human Airway Epithelia at the Air–Liquid Interface (ALI)

The 3D airway epithelia (MucilAir^™^, Epithelix, Geneva, Switzerland) reconstituted in vitro using primary small airways human cell were fully differentiated and functional, using similar conditions previously reported [32,33,34]. The cells were received in 24-well Transwell inserts^®^ in a gel matrix. Once the cells were received, they were transferred into a new 24-well plate with 700 µL of the MucilAir™ media in the basal surface. Media was changed every other day.

After 3 days of incubation at 37 °C and 5% CO_2_, experiments were performed. For in vitro cell dose response, the cells were exposed to different concentrations of the drug formulation dissolved in 90:10 media: ethanol to facilitate dissolution. After 72 h of incubation, the inserts were rinsed with a 6 µM Resazurin solution in order to eliminate the remaining red phenol from the cell growth media. The inserts were transferred to a new 24-well plate filled will 500 µL/well of Resazurin solution and then 200 µL/well was added in the apical surface. After one hour of incubation, 100 µL from the apical side were transferred to a 96-black well plate. At this point, the fluorescence intensity of the resorufin (fluorescent metabolite) produced by viable cells was detected at 544 nm (excitation) and 590 nm (emission) using the Synergy H1 Multi-Mode Reader (BioTek Instruments, Inc., Winooski, VT, USA). The relative viability of cell line was calculated with Equation (6). This protocol was provided by the vendor [35].

#### 2.2.19. In Vitro Transepithelial Electrical Resistance (TEER) of 3D Human Airway Epithelia at the ALI

The TEER values were measured using similar conditions previously reported [32,33,34] and the vendor protocol [53]. After receiving the inserts, the cells were moved into a fresh 24 well plate pre-filled with 700 µL of MucilAir^™^ media in the basal side. The experiments were done after 3 days of incubation. Briefly, 100 µM of PNA5 and co-SD PNA: Trehalose dissolved in PBS: Ethanol were added to the apical side of the inserts, so the cells were exposed to the formulations. TEER values were obtained before exposure to the drug solutions and after exposure to them (3 h) and then every 24 h for seven days. To measure TEER values, the EVOMX (Epithelial Volt/Ohm Meter) and electrode (STX2) (World Precision Instruments, Sarasota, FL, USA) were used. A total of 200 µL of the cell media were added to the apical surface of the inserts. The prolonged part of the electrode was immersed through the gap of the insert and tilted on the bottom of the well, and the short stem was above in the apical surface, inside the culture media. After each measurement, the media was removed from the apical surface to leave the cells at ALI conditions.

## 3. Results

### 3.1. Solid Phase Peptide Synthesis (SPPS)

**Figure 2:**
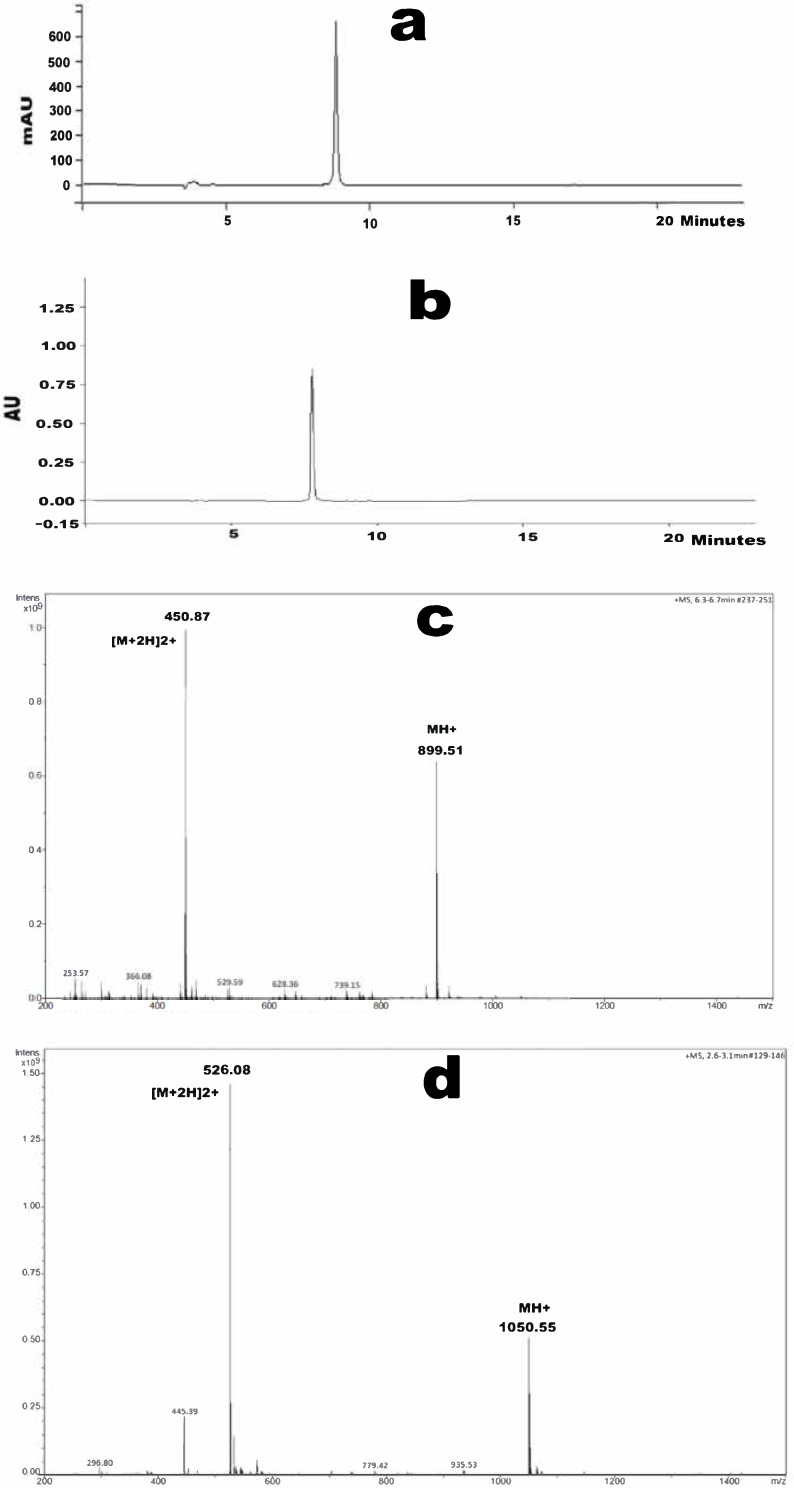
The purity and mass measurements of Ang (1—7) and PNA5. (**a**) Analytical HPLC chromatogram of pure Ang (1—7). (**b**) Analytical HPLC chromatogram of pure PNA5. (**c**) Mass spectroscopy spectrum of pure Ang (1—7) shows single charged ion ([MH]^+^: 899.51) and double charged ion ([M+2H]^2+^: 450.87). (**d**) Mass spectroscopy spectrum of pure PNA5 shows single charged ion ([MH]^+^: 1050.55) and double charged ion ([M+2H]^2+^: 526.08).

One gram of both Ang (1—7) and PNA5 were successfully synthesized and characterized. Synthetic details for Ang (1—7) and its glucoside PNA5 have been previously reported [15,22]. Preparative HPLC was used to purify the crudes producing one gram of each compound with a purity of more than 98.5% confirmed by analytical HPLC as shown in (Figure 2a,b) for Ang (1—7) and PNA5, respectively. The retention time of Ang (1—7) is 8.80 min and 7.74 min for PNA5. Chemical composition and molecular weight for both compounds were performed after purification using mass spectroscopy and proved the successful synthesis and purification of the targeted compounds. For Ang (1—7) (C_41_H_62_N_12_O_11_), the molecular weight was 899.02 g/mol and the exact mass was 898.47 g/mol (Figure 2c), which showed the mass spectra for Ang (1—7). The molecular weight for PNA5 (C_45_H_71_N1_3_O_16_) was 1050.14 g/mol and the exact mass was 1049.51 g/mol (Figure 2d), which showed the mass spectra of PNA5.

### 3.2. Computational Predictions of Physicochemical Parameters of Ang (1—7) Peptide and PNA5 Glycopeptide

**Figure 3:**
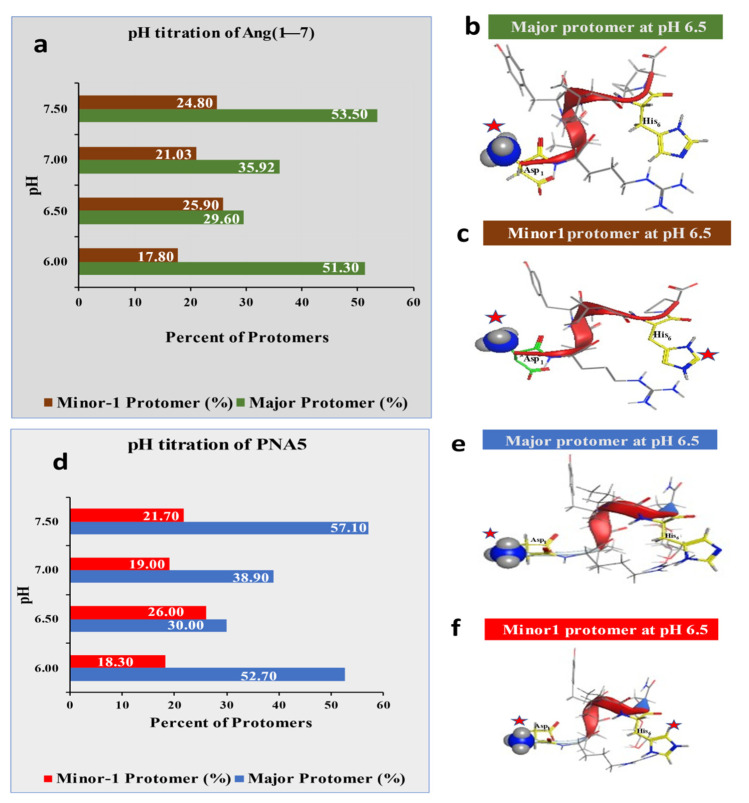

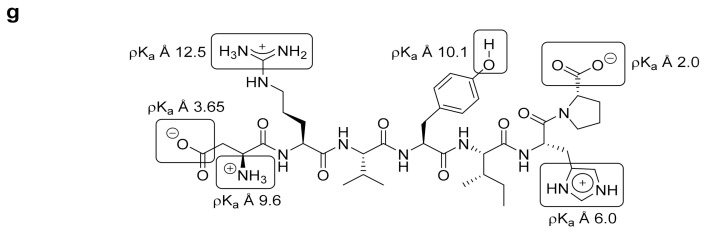
Pseudo-isoelectric point determination using MOE software. (**a**) Computational pH titration of Ang (1—7) with protomers percentages at various pHs. (**b**) Ang (1—7) major protomer 3D-structure; (**c**) Ang (1—7) minor-1 protomer 3D-structure. (**d**) Computational pH titration of PNA5 with protomers percentages at various pHs. (**e**) PNA5 major protomer 3D-structure; (**f**) PNA5 minor-1 protomer 3D-structure. (**g**) Ang (1—7) structure marked with pKas of the groups contributing to the polarity of various protomers under different pHs. * The red stars on the structures marked the positively charged groups.

In this study, the primarily focused descriptors related to pH titration, protomers population, solubility (h_LogS), and lipophilicity (h_LogP). Computational pH titrations of Ang (1—7) and PNA5 using MOE software were performed (Figure 3). These titrations helped determine proper pH, resulting in a pseudo-isoelectric point with lower solubility (less charged) of the compounds in an aqueous environment and higher solubility in organic solvents (alcohols). Since adjusting the pH to the isocratic point is essential for optimizing the solubility and lipophilicity experiments to suppress the ionizable groups.

In Ang (1—7), there are six different ionizable groups with six different pKas, as labeled in (Figure 3g), and for PNA5, there are five different pKas. Since the pH range under study is from 6 to 6.5, the Tyr^4^ (p-OH) and Arg^2^ (δ-guanidine) groups (yellow colored) will retain their neutral and positive charge because of their pKa values are out of the pH range. On the other hand, Asp^1^ (β-COOH and Pro^7^-C-terminal–COOH) (gray colored) stay negatively charged over the used pH range. Only two functional groups, His^6^ imidazole, and Asp^1^-α-NH_2_ (green colored) are the leading players in defining the effective charges of the molecules. pH 6.5 was selected as a pseudo isoelectric point for Ang (1—7) and PNA5 due to more than 62% neutral His6 (no charged imidazole π-N group), 40% neutral Asp^1^-α-NH_2_, and less than 60% Asp^1^-α-NH^+^_3_charged. These numbers are better than the numbers determined for Ang (1—7) and PNA5 at pH 6 and 6.2. Computational solubility and logP are included in the solubility and partition (logP) sections.

### 3.3. Solubility

Using the computational approach, pH 6.5 was selected to study the solubility behavior of Ang (1—7) and PNA5 experimentally in various solvents. As shown in Table 2, the experimental measurements of Ang (1—7) and PNA5 solubilities in water, PBS, and NS are in the range of 100–1000 mg/mL, the freely soluble category according to the USP solubility definitions. Ang (1—7) was slightly soluble in methanol and very slightly soluble in ethanol. PNA5 was found to be very slightly soluble in both methanol and ethanol. The effect of the sugar moiety in PNA5 increases its solubility of PNA5 in the aqueous solvents while decreasing its solubility in the organic solvents compared to native Ang (1—7). Ang (1—7) experimental LogS is −0.71 while for the computational h_LogS of −0.84 using MOE, and for PNA5, both the experimental and computational values were much closer of −0.58 (LogS) and −0.53 (h_LogS), respectively. This result gave high confidence in our experimental determination of solubility. The computational methodology for solubility is based on applying the Hückel theory to build theoretical solubility determination methods of various compounds [54].

### 3.4. Partitioning Study (LogP)

We used practical and computational approaches to determine the lipophilic properties of Ang (1—7) and PNA5. In the experimental method, SFM was used to evaluate n-octanol/aqueous logP at room and physiological temperatures. After carrying the experiment, samples from organic and aqueous layers were injected into the HPLC using a well-developed analytical method. The chromatograms showed a large sharp peak of both compounds in the aqueous layer, while no compound peaks were detected in the organic layers (analytical chromatograms are not shown here). Thus, the Ang (1—7) and PNA5 concentrations in the organic layer were below the detection limits of the instrument and, it was hard to detect them using this analytical HPLC method. However, this indicates the high hydrophilicity of these two compounds. In the computational approach, MOE software was used to calculate the descriptor of h_LogP to compare it to the experimentally determined LogP. As the experimental LogP of Ang (1—7) and PNA5 was undetermined, we used the computational approach, which allowed us to look at the results relative to the level of the protomers in the solution (Figure 3). The aqueous medium was dictated by the compounds’ ionization status, which reflected their distribution in the two layers of the immiscible solvent components. The results showed h_logP of −9.2 for Ang (1—7) and h_logP of −12.0 for PNA5, which parallel with both compounds’ expected hydrophilicity.

### 3.5. Scanning Electron Microscopy (SEM)

**Figure 4:**
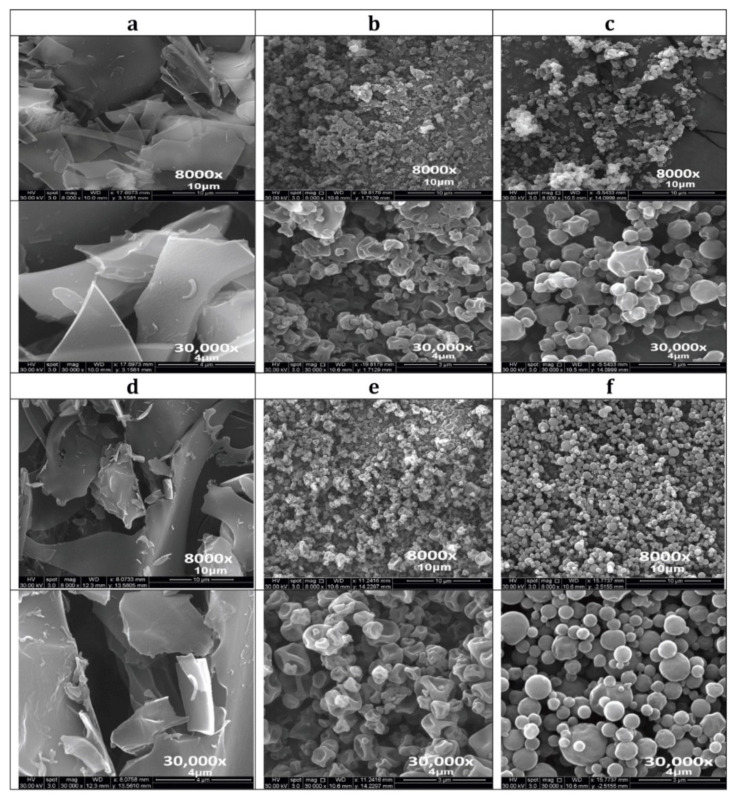
SEM micrographs at two magnifications (8000× & 30,000×) of each sample: (**a**) Raw Ang (1—7) (unprocessed); (**b**) SD Ang (1—7); (**c**) Co-SD Ang (1—7):Trehalose (25:75); (**d**) Raw PNA5 (unprocessed); (**e**) SD PNA5; and (**f**). Co-SD Ang PNA5:Trehalose (25:75).

SEM investigated the particle surface morphology, particle shape, and particle size of the: raw non-SD Ang (1—7), PNA5, Trehalose, SD Ang (1—7), SD PNA5, co-SD Ang (1—7): Trehalose, and co-SD PNA5: Trehalose formulations. (Figure 4) shows the SEM micrographs at two magnifications, and Table 3 shows the particle size diameter, standard deviation, and range as quantified statistically using SigmaScan Pro5.0.0 (Systat, San Jose, CA, USA) software. The spray drying process significantly affected the final powder properties, such as particle shape, particle morphology, particle surface morphology, and particle size. Chunk-like powders of raw Ang (1—7) (Figure 4a) and raw PNA5 (Figure 4d) were produced, while small-shriveled particles for SD Ang (1—7) (Figure 4b) and SD PNA5 (Figure 4e). Using Trehalose as an excipient in the co-SD two-component system (Co-SD Ang (1—7):Trehalose (25:75) (Figure 4c) and Co-SD PNA5:Trehalose (25:75) (Figure 4f) produced powders with more favorable properties, such as smaller-spherical particles with smoother surface morphology. For particle sizing and size distribution by image analysis of SEM micrographs, a representative micrograph for each sample (Figure 4. top images at 8000× magnification) was analyzed by measuring the diameter of at least 100 particles per sample. As shown in Table 3, less than 1.00 μm particle size for the SD one-component system powders. On the other hand, around 0.50 μm for the co-SD two-component system powders (co-SD Ang (1—7):Trehalose (25:75), and co-SD PNA5:Trehalose (25:75)) with narrower size distribution rang.

**Figure 5:**
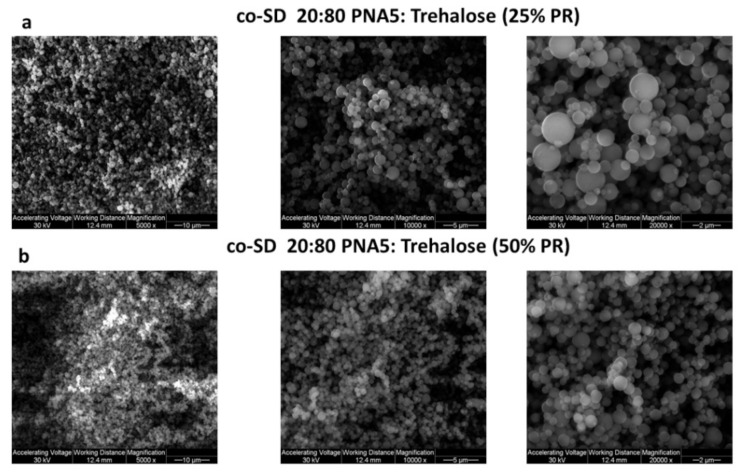
SEM micrographs at three magnifications (5000, 10,000× & 20,000×) of each sample: (**a**) co-SD PNA5: Trehalose (20:80) 25% PR, (**b**) co-SD PNA5: Trehalose (20:80) 50% PR.

**Figure 6:**
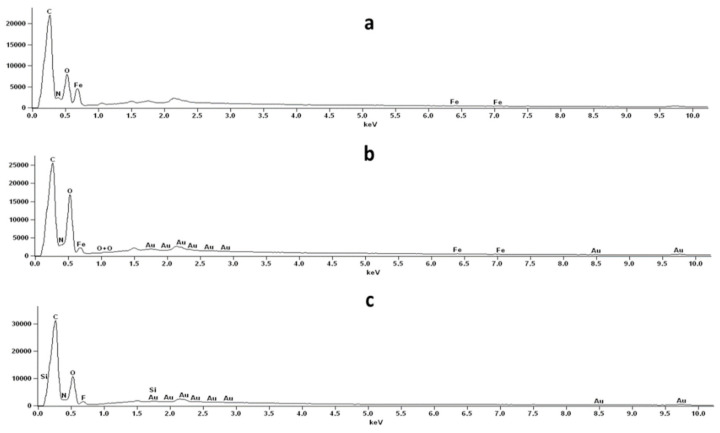
SEM-EDX graph of (**a**) Raw Trehalose (**b**) co-SD PNA5: Trehalose (20:80) 25% PR, (**c**) co-SD PNA5: Trehalose (20:80) 50% PR.

Figure 5 shows the SEM micrographs of SEM micrographs at three magnifications (5000, 10,000× & 20,000×) of co-SD PNA5: Trehalose (20:80) at 25% PR, and co-SD PNA5: Trehalose (20:80) at 50% PR. SEM-EDX data showed the presence of the Nitrogen atom, which is abundant in PNA5 (Figure 6).

### 3.6. X-ray Powder Diffraction (XRPD)

**Figure 7:**
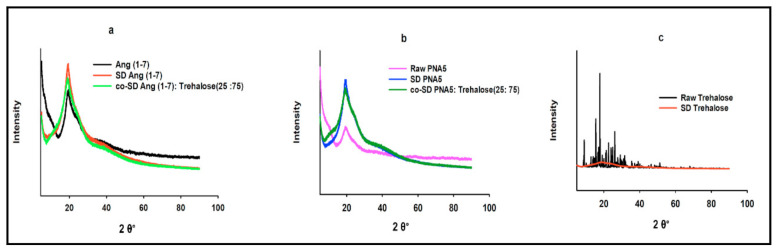
XRPD diffraction patterns for: (**a**) Raw Ang (1—7), SD Ang (1—7), co-SD Ang (1—7):Trehalose (25:75); (**b**) Raw PNA5, SD PNA5, co-SD PNA5:Trehalose (25:75); (**c**) Raw Trehalose, SD Trehalose.

**Figure 8:**
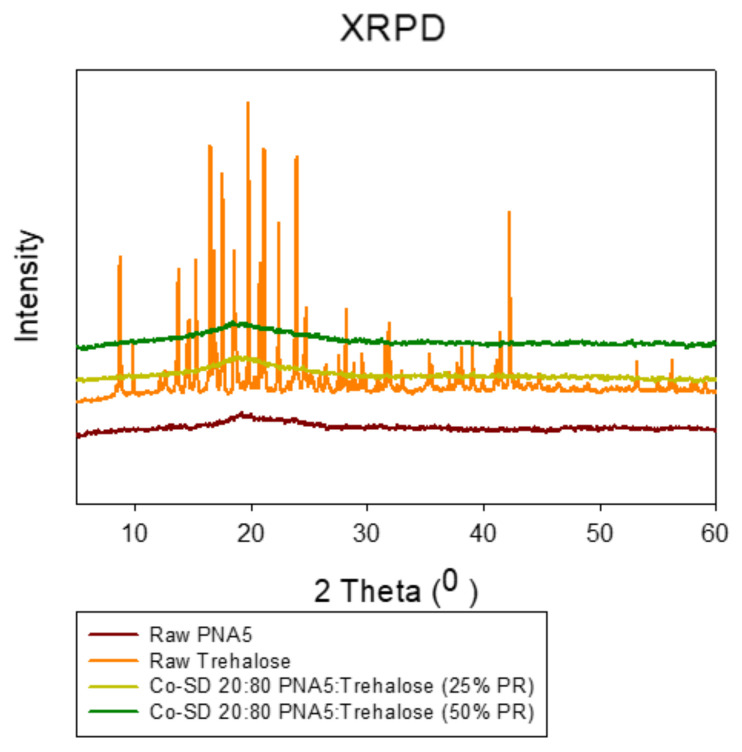
XRPD diffraction patterns for: Raw PNA5 (1—7), Raw Trehalose, co-SD PNA5: Trehalose (20:80) 25% PR, co-SD PNA5:Trehalose (20:80) 50% PR.

The crystallinity of the raw Ang (1—7), raw Trehalose, all SD and co-SD formulations were examined by studying its XRPD. As shown in (Figure 7 and Figure 8), all powders’ XRPD diffractogram patterns except for raw Trehalose are without any characteristic crystalline peaks. They indicated the lack of long-range molecular order, which was consistent with non-crystalline powders exhibiting amorphous character. In contrast, as supplied by the manufacturer, the raw Trehalose was characterized by having an XRPD diffractogram pattern with multiple sharp peaks, which are characteristic of the long-range molecular order present in crystalline materials. Following organic solution spray drying, these distinct peaks were no longer present, suggesting the loss of crystallinity for SD Trehalose and all co-SD two-component system powders.

### 3.7. Differential Scanning Calorimetry (DSC)

**Figure 9:**
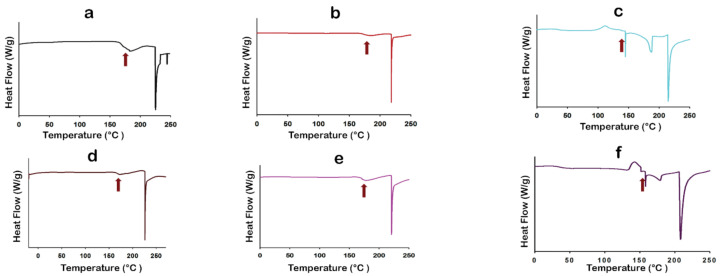

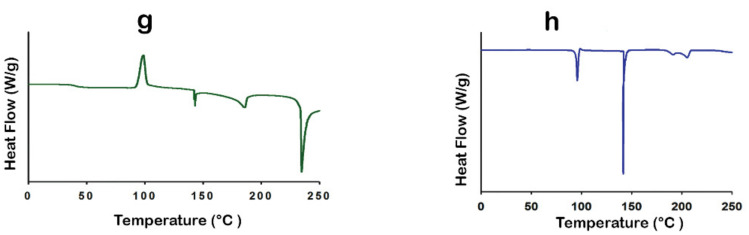
Differential scanning calorimetry thermograms of (**a**) Raw Ang (1—7); (**b**) SD Ang (1—7); (**c**) co-SD Ang (1—7):Trehalose (25:75); (**d**) Raw PNA5; (**e**) SD PNA5; (**f**) co-SD PNA5:Trehalose (25:75); (**g**) SD Trehalose; (**h**) Raw Trehalose. The red arrows marked the T_g_.

Thermal analysis of raw components, SD single component, and co-SD binary components particles was done by the DSC technique. (Figure 9) shows a representative DSC thermogram for each powder at 5.00 °C/min heating rate. Table 4 summarizes the thermal analysis of all DSC thermograms. Raw Ang (1—7), raw PNA5, SD single-component, and co-SD two-component thermograms show clear and high glass transition temperature (T_g_) values (second-order solid-state phase transition from the amorphous glass to the amorphous rubber) at a range of ~165–180 °C. All these powders exist in an amorphous state before and after spray drying. The T_g_ values were significantly above room temperature. Hence, the powders were in the amorphous glassy state at room temperature, consistent with the XRPD patterns. The DSC thermograms at 40.0 °C/min heating rate showed relatively close Tg values to the ones at 5.00 °C/min heating rate (thermograms are not shown).

Raw Ang (1—7), raw PNA5, SD Ang (1—7), and SD PNA5 thermograms exhibit a single endotherm representing the melting point at a range of ~217–232 °C. While the co-SD binary system of Ang (1—7) and PNA5 with Trehalose exhibited two endothermic peaks ~169–185 °C and the other one is ~204–213 °C, beside an exothermic peak ~115 °C for co-SD Ang (1—7):Trehalose (25:75) powder, and ~143 °C for co-SD PNA5: Trehalose (25: 75) powder. All these transitions in the co-SD binary systems indicate a good encapsulation of the Ang (1—7) and PNA5 in the trehalose during the spray drying process since these transitions exist in the SD Trehalose thermogram also. The presence of Trehalose in the co-SD systems appeared to decrease the T_g_ values for the binary co-SD system. However, the T_g_ for these powders is still significantly above room temperature and physiological temperature, and this decrease in T_g_ is not expected to affect the binary powders’ stability.

The thermograms obtained from raw trehalose and SD trehalose are shown in (Figure 9g,h). Three endothermic peaks for raw trehalose are characteristic for order-to-disorder thermotropic phase transitions. The two endothermic order-to-disorder peaks at ~96 °C and 141 °C correspond to two dehydration processes due to the loss of unbound water at ~100 °C (i.e., vaporization of unbound water), vaporization of the bound water molecules at 141 °C. The third endothermic order-to-disorder peak at 204 °C represented the fusion of anhydrous β form of trehalose. SD Trehalose showed an amorphous glassy-to-rubbery phase transition T_g_ (i.e., characteristic baseline shift) at ~41 °C, followed by an exothermic disorder-to-order phase transition near 100 °C, which suggests crystallization at T_c_ from the amorphous rubbery state. A small endothermic order-to-disorder peak was observed in the temperature range of ~146 °C. Similar DSC thermograms were obtained for raw and SD Trehalose powders [24,55]. All DSC data agree with the XRPD data, and the degradation for all powders occurred at a temperature higher than 250 °C.

### 3.8. HSM under Cross-Polarizer Lens

**Figure 10:**
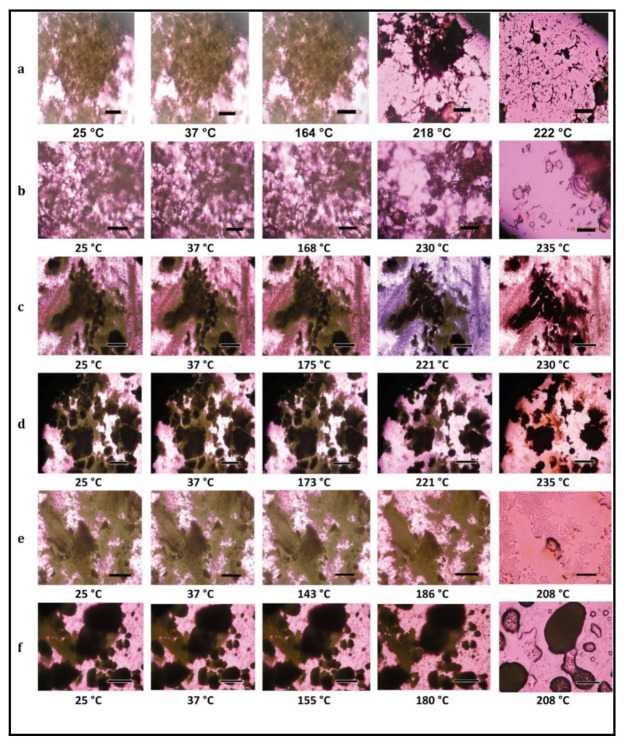
Representative HSM images for: (**a**) Raw Ang (1—7); (**b**) Raw PNA5; (**c**) SD Ang (1—7); (**d**) SD PNA5; (**e**) co-SD Ang (1—7):Trehalose (25:75); (**f**) co-SD PNA5:Trehalose (25:75). Scale bar: 10 μm.

HSM at a rate of 5 °C/min for raw Ang (1—7), raw PNA5, SD Ang (1—7), SD PNA5, co-SD Ang (1—7):Trehalose (25:75), and co-SD PNA5:Trehalose (25:75) showed dark agglomerates lack birefringence over the whole temperature range, i.e., the powders’ amorphous character, which confirms the XRPD, and DSC data. One observable thermal event of melting (i.e., an order-to-disorder phase transition) from the solid-state to the liquid state for raw Ang (1—7) (Figure 10a) and raw PNA5 (Figure 10b), which began to liquefy by melting at 218 °C and 230 °C, respectively (melting point). SD Ang (1—7) (Figure 10c) and SD PNA5 (Figure 10d) started to melt at the same temperatures as their raw counterparts; still, they did not visually complete melting before the degradation occurred at a temperature above 250 °C; this may be due to these two formulations’ small particle sizes. For the co-SD Ang (1—7):Trehalose (25:75) powder (Figure 10e), and co-SD PNA5:Trehalose (25:75) powder (Figure 10f), the only visualized transition was the melting that occurred at ~208 °C, so the exotherm peak and the other two endotherms seen in their thermograms were not visually evident in HSM, which also may be due to the small particle size ~0.5 μm (Table 3) of these two formulations.

Raw Trehalose (micrographs are not shown here) was highly crystalline due to extensive birefringence, which started to diminish upon heating at temperature ~100 °C and finished at 105 °C, is the first endothermic change vaporization of unbound water. Interestingly, the particle shape remained at this transition and started to collapse at a higher temperature during the second endothermic order-to-disorder phase transition. At the range 146–155 °C, the crystal shape disappeared at that temperature range. Birefringence appeared again in the temperature range of 185–200 °C, suggesting a subtle recrystallization event. Melting was visualized, starting at the temperature range of 200–213 °C by forming liquid droplets. SD Trehalose showed dark agglomerates lacking birefringence (micrographs are not shown here), which indicated an amorphous. The recrystallization of SD Trehalose with increasing temperature was not visualized. The first phase transition visualized by HSM appears to correlate with the small order-to-disorder endotherm (due to a metastable phase transition), as observed in the DSC thermogram at ~145 °C; the melting process occurred at higher temperatures. The decomposition for all samples occurred at a temperature above 250 °C. All results are in good agreement with the DSC thermograms for all powders visualized by HSM.

### 3.9. Karl Fisher Titration (KFT)

The residual water content values for all powders were analytically quantified by KF and listed in Table 5. The water content values were lower than counterparts’ powders for all SD and co-SD powders, indicating that water was removed following organic solution advanced spray drying in closed mode from a methanol solution. The mean total water content result of raw Trehalose is in excellent agreement with the literature [24,55,56], which gives confidence in our data. All water content values remained relatively low, which is significantly crucial for chemical and formulation stability.

### 3.10. ATR-FTIR Spectroscopy

**Figure 11:**
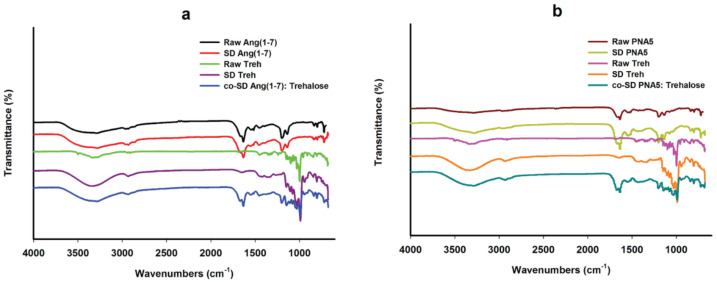
ATR-FTIR spectra of (**a**) Raw Ang (1—7), SD-Ang (1—7), Raw Trehalose, SD Trehalose, co-SD Ang (1—7):Trehalose (25:75); (**b**) Raw PNA5, SD PNA5, Raw Trehalose, SD Trehalose, co-SD PNA5:Trehalose (25:75).

Infrared spectroscopy is a useful analytical technique to determine the presence of functional groups in molecules. It provides spectral data regarding any change in a compound molecule’s functional group characteristics while processing a formulation and after spray drying. FTIR spectra are shown in (Figure 11). FTIR spectra of raw Ang (1—7) and raw PNA5 are nearly the same; one prominent characteristic peak was found between 3650 and 3200 cm^−1^, which was assigned to the stretching vibration of the OH group and intramolecular hydrogen bonding. The small peak at 2900 cm^−1^ assigned to C-H. The band at ~1700–1650 cm^−1^ represented the acidic carbonyl C=O stretching. The little peak at ~1550 to 1500 cm^−1^ represented the CH2 of the aromatic ring. The peak at 1370 cm^−1^ represented the bending vibration of the hydroxyl group. The band at 1250–1050 cm^−1^ was assigned to C-O stretching vibrations. These characteristic peaks also appeared in the SD Ang (1—7) and SD PNA5 spectra; thus, the spray drying process did not cause any change in the functional groups of Ang (1—7) and PNA5.

The FTIR spectra of raw and SD trehalose showed a characteristic peak at 3650 cm^−1^ to 3000 cm^−1^, assigned to the OH group’s stretching vibration intramolecular hydrogen bonding. Another peak at 2900 cm^−1^ assigned to C-H. A strong band at 1250 to 900 cm^−1^ was assigned to C-O. ATR-FTIR spectra of co-SD Ang (1—7):Trehalose (25:75) (Figure 11a), and ATR-FTIR spectra of co-SD PNA5:Trehalose (25:75) (Figure 11b) confirm the presence of both components in the co-SD particles, i.e., the presence of the same peaks, which are characteristic functional groups of pure Ang (1—7) and trehalose in the co-SD Ang: Trehalose powder, and the presence of characteristic functional groups of pure PNA5 and Trehalose in the co-SD PNA5: Trehalose powder.

### 3.11. Confocal Raman Microspectroscopy (CRM)

**Figure 12:**
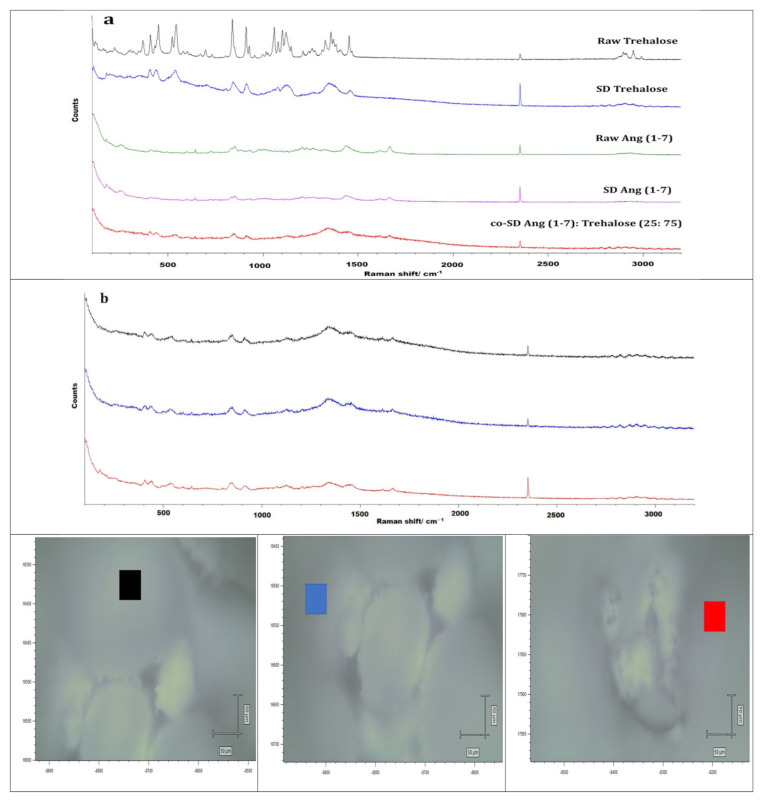
Raman spectra of (**a**) Raw Trehalose, SD Trehalose, Raw Ang (1—7), SD Ang (1—7), and co-SD Ang (1—7): Trehalose (25: 75). (**b**) Raman spectra for co-SD Ang (1—7):Trehalose (25:75) at three spots with the powder’s microscopic images in each spot. Scale bar: 50 μm.

**Figure 13:**
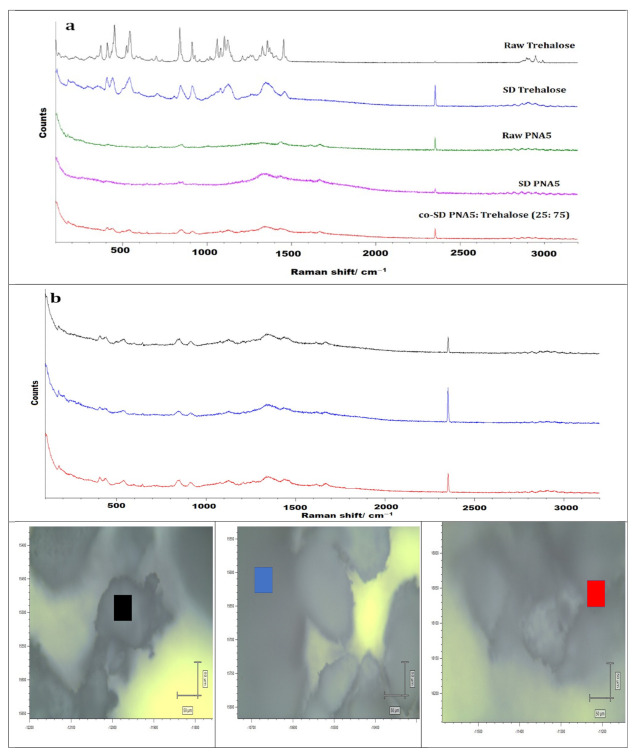
Raman spectra of (**a**) Raw Trehalose, SD Trehalose, Raw PNA5, SD PNA5, and co-SD PNA5:Trehalose (25:75). (**b**) Raman spectra for co-SD PNA5:Trehalose (25:75) at three spots with the powder’s microscopic images in each spot. Scale bar: 50 μm.

**Figure 14:**
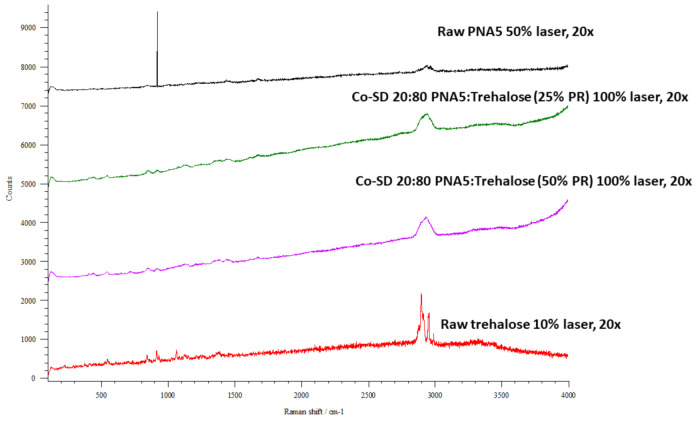
Raman spectra of Raw PNA5, co-SD PNA5: Trehalose (20:80) 25% PR; co-SD PNA5: Trehalose (20:80) 50% PR; Raw Trehalose.

Raman microscopy analysis was performed to check the chemical composition, the physical form, and all powders’ homogeneity; therefore, a spectral scan from 100–4000 cm^−1^ was performed. All the samples exhibited no crystallinity before and after spray drying except raw trehalose, which showed high crystallinity before processing but no crystallinity after spray drying. These were in good agreement with DSC, XRPD, and HSM. Figure 12, Figure 13 and Figure 14 exhibit the characteristic peaks corresponding to Raman analysis based on the spectral scan of both raw components and their SD and co-SD powders. Raw Ang (1—7) and raw PNA5 exhibited the same Raman spectra as their SD counterparts. Many Raman shifts were observed. The first one was a weak peak in the range of 2800–3300 cm^−1^ in all powders. A 2300 to 2400 cm^−1^ peak was observed in all powders and related to the silicon wafer used in the experiments. A Raman shift at 1300 to 1400 cm^−1^ in raw Ang (1—7), raw PNA5, their SD, and co-SD counterpart’s powders. Two peaks at 850 cm^−1^ and 950 cm^−1^ appeared in raw trehalose and its SD and co-SD powders. The spectra showed the presence of both components in the co-SD particles, i.e., the characteristic functional groups of pure Ang (1—7) and trehalose in the co-SD Ang: Trehalose powder. Moreover, the presence of characteristic functional groups of pure PNA5 and Trehalose in the co-SD PNA5: Trehalose powders (Figure 13 and Figure 14) agreed with ATR-FTIR data.

Co-SD samples (co-SD Ang (1—7):Trehalose (25:75) and co-SD PNA5: Trehalose (25: 75) appear to exhibit homogeneity; this was probed by measuring three different locations on each sample. (Figure 12b and Figure 13b) show representative brightfield micrographs obtained at 20× magnification of co-SD samples and the corresponding Raman signal obtained from different regions of the imaged sample. Each colored square in each image represents the powder’s specific spot was used to assess the chemical composition, and the corresponding spectra are shown (same color of the square). The peaks in the three spectra for each powder from different spots are consistently seen, suggesting a uniform distribution of the components.

### 3.12. In Vitro Aerosol Dispersion Performance

**Figure 15:**
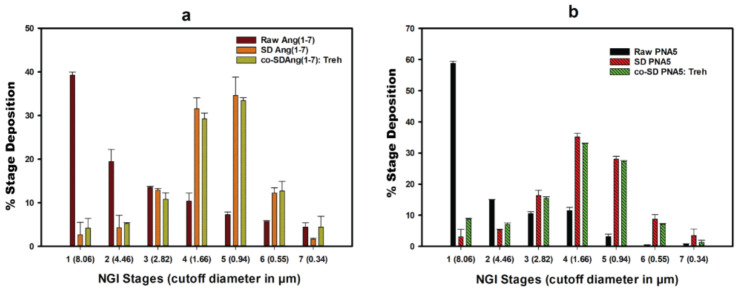
In vitro aerosol dispersion performance as DPIs using the NGI and the human DPI device, the Neohaler^®^ for: (**a**) Raw Ang (1—7), SD-Ang (1—7), and co-SD Ang (1—7):Trehalose (25:75); (**b**) Raw PNA5, SD PNA5, and co-SD PNA5:Trehalose (25:75).

Figure 15 shows in vitro aerosol dispersion performance profiles using NGI^®^ at Q = 60 L/min. All powders performed as dry powder aerosols with measurable aerosol deposition on all NGI stages, including the lowest NGI stage, stage 7. Raw Ang (1—7), raw PNA5 exhibited high particle deposition on stages 1–3 and low particle deposition on stages 5–7. On the other hand, the spray drying process for all SD and the inclusion of Trehalose in the co-SD systems had a profound effect on the stage deposition. As can be seen in (Figure 15), SD Ang (1—7), SD PNA5, co-SD Ang (1—7):Trehalose (25:75), and co-SD PNA5:Trehalose (25:75) systems decreased the deposition on stages (1–3) but increased the aerosol deposition on stages 4, 5, and 6 that include solid-state nanoparticles. The aerosol dispersion performance parameter values are shown in Table 6. Raw Ang (1—7) and raw PNA5 have higher ED% than their SD and co-SD counterparts; still, the ED% of all processed powders are considered high (64–86%). The FPF% of all SD and co-SD systems were significantly improved than the raw counterparts’ powders. Moreover, the MMAD of less than 2.09 μm from the Neohaler^®^ device at Q = 60 L/min for all SD and co-SD powders compared with significantly higher MMAD of raw Ang (1—7) and raw PNA5. The GSD decreased, indicating the aerodynamic size distribution became narrower in the SD and co-SD powders compared with the GSD of the raw powders.

### 3.13. In Vitro 2D Cell Viability of Human Cells

**Figure 16:**
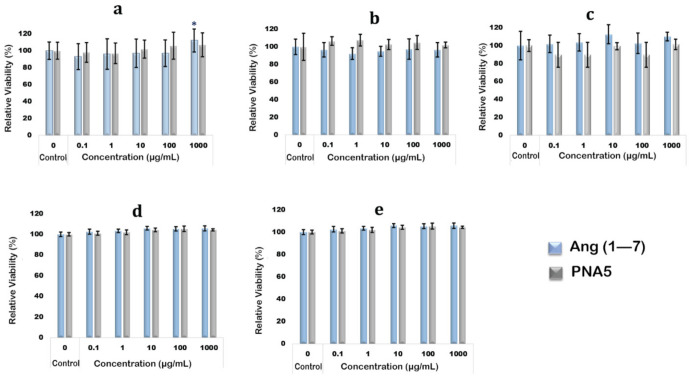
In vitro cell viability (*n* = 6, mean ± SD) using resazurin assay after exposure to different concentrations of Ang (1—7) and PNA5 for (**a**) RPMI2650; (**b**) NHA; (**c**). hCMEC/d3; (**d**) A549; and (**e**) H441 cell lines.

Human cells from the organs expected to be exposed to Ang (1—7) peptide and PNA5 glycopeptide upon treatment were used to test the compounds’ biocompatibility and safety in the context of cell viability. Nasal cells (RPMI 2650), brain cells (NHA and hCMEC/D3), and lung cells (A549 and H441) were successfully cultured in the suitable supplemental media as mentioned in the method section. Cell monolayer development was achieved within each cell line’s expected time. Seeding the cells in 96-well plates and treating them with compounds were done successfully. For all tested cell lines, the resazurin assay for cell viability experiments showed no statistically significant difference in relative cell viability% between the control cells (not treated with compound) and the cells exposed to the Ang (1—7) and PNA5 at concentrations of 0.1 µg/mL, 1 µg/mL, 10 µg/mL, and 1000 µg/mL (*p* values > 0.05). It revealed that both compounds did not significantly affect cell viability of human nasal epithelium RPMI2650 cells (Figure 16a; astrocyte brain cells (NHA) (Figure 16b); endothelial brain cells hCMEC/D3 (Figure 16c); A549 lung epithelial cells (Figure 16d), and H441 lung epithelial cells (Figure 16e).

### 3.14. In Vitro Cell Viability of 3D Human Airway Epithelia

**Figure 17:**
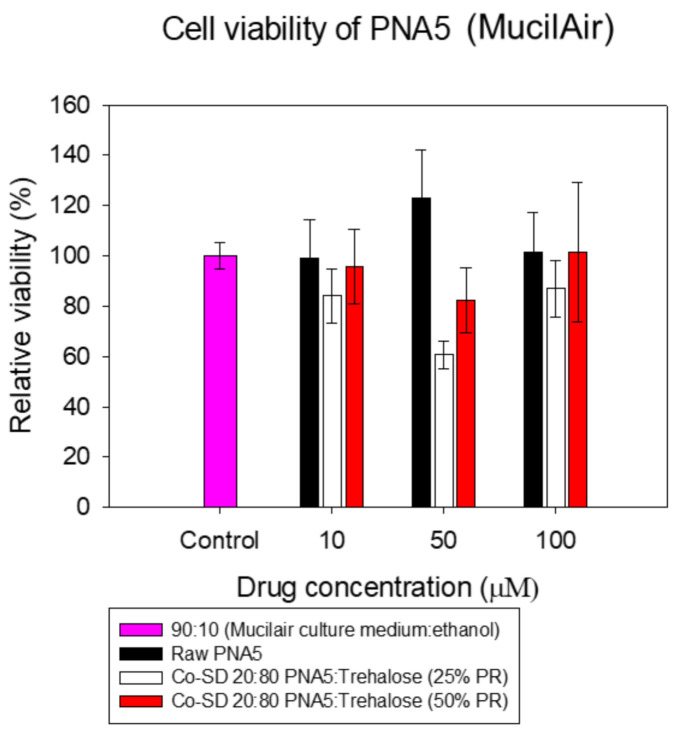
In vitro cell viability (*n* = 3, mean ± SD) using resazurin assay after exposure to different concentrations of PNA5 and Co-SD PNA5: Trehalose at 25% PR and 50% PR for MucilAir^®^ 3D cell line.

As shown in Figure 17, all formulations maintained the high cell viability of MucilAir^®^ cells after 72 h of exposure at different concentrations.

### 3.15. In Vitro 2D TEER at the ALI

**Figure 18:**
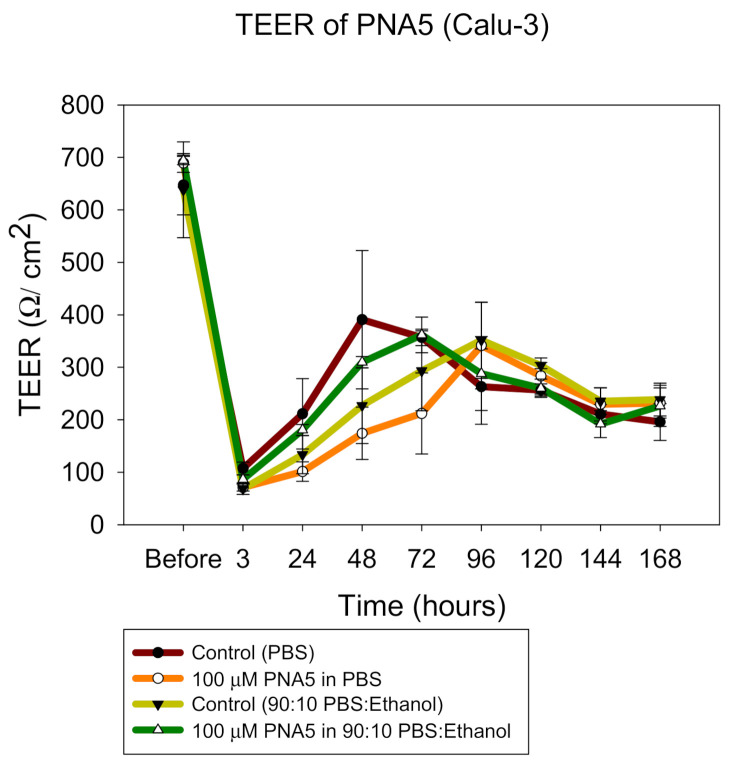
In vitro TEER response (*n* = 6, mean ± SD) to 100 µM of PNA5 in different solvents systems.

TEER measurements were successfully performed on CaLu-3 cells at ALI conditions to determine the effect of the SD particles on the cell monolayer. The existence of a complete monolayer at ALI was confirmed by TEER values more than 500 Ω/cm^2^ after 7 days of exposure and by the observance of the monolayer via light microscopy (data not shown). As shown in Figure 18, after 3 h of exposure, TEER values dropped significantly; however, after seven days of culturing, it was seen that TEER values were ~500 Ω/cm^2^, which is the normal value for Calu3 cells in ALI conditions.

### 3.16. In Vitro TEER 3D Human Airway Epithelia at the Air–Liquid Interface (ALI)

**Figure 19:**
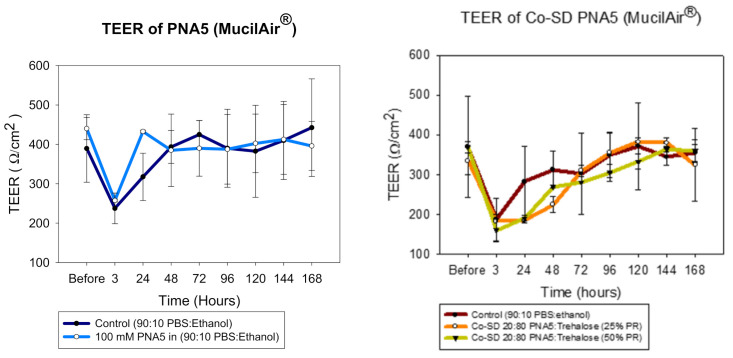
In vitro TEER response (*n* = 3, mean ± SD) to 100 µM of PNA5 (left graph) and to 100 µM of co:SD PNA5: trehalose (20:80) at 25% PR and 50% PR (right graph).

The same trend was observed when MucilAir^®^ cells were exposed to the formulations (Figure 19). The TEER values dropped significantly right after the application; however, within a few days, the monolayer was completely recovered, consistent with an earlier reported study by our group [46].

## 4. Discussion

To the authors’ knowledge, this study is the first to use and report this approach to rationally design and produce inhalable dry powders of Ang (1—7) and its glycosylated derivative PNA5 for respiratory delivery. These two compounds were successfully synthesized using the SPPS strategy with high purity, then formulated as dry powders. The novel particles were produced by organic solution advanced spray drying from dilute solution with rationally chosen spray drying conditions. Additionally, a comprehensive characterization of the physiochemical properties of the powders before and after formulation was performed.

The solubility and lipophilicity (logP) are the most crucial properties that affect drug release pharmacokinetics. Thus, the determination of such pharmaceutical properties is essential in drug research’s early stages [41]. In this study, the solubility and logP of Ang (1—7) and PNA5 were determined experimentally and computationally; the results from both approaches were very close and showed that these two compounds are very hydrophilic. PNA5 is an improved version of Ang (1—7); it is an amphipathic glycopeptide that contains a carbohydrate moiety. The presence of this moiety affects its interactions with the biological membranes. As expected, the sugar moiety in the PNA5 increased its aqueous solubility; hence, its overall hydrophilicity compared with Ang (1—7), as can be concluded from the computational logP values. In general, glycopeptides have relatively lipophilic backbones and hydrophilic sugar moieties; this allows a glycopeptide to interact with both the membrane surface and aqueous compartment in a "hopping" motion, which promotes transport of the drug throughout the body in vivo and improves the bioavailability [15,18,21,57]. Thus, these solubility and lipophilicity studies give important information, to expect the in vivo biodistribution, in terms of glycopeptides’ biousian character (two essences). This biousian character was suggested by Polt and coworkers, referring to the two different conformational states the glycopeptide can adopt in the aqueous bulk compartment and when bound to the membrane [15].

The surface morphology, as visualized by SEM, changed after formulation. The raw Ang (1—7) and PNA5 were large chunks before processing. A different morphology after the spray drying process, specifically corrugated and smooth particles, was produced for SD pure compounds and co-SD two-component systems, respectively. The non-aqueous nature and inherently lower surface tension of an organic solvent combined with a low (dilute) solute concentration enable particle production in the solid-state down to the inhalable nanometer size range with maintaining a narrow particle size distribution in the solid-state. The SEM images’ particle size analysis showed that a small average particle size down to less than 900 nm and 500 nm for SD powders and co-SD powders, respectively, was achieved. The SEM-EDX was extremely helpful in confirming the presence of PNA5 in the dry powders. It was clearly seen the presence of Nitrogen which is very prominent in the molecule.

In this study, Trehalose was used as an excipient in the binary co-SD systems. Our group successfully used this excipient to formulate dry powders suitable for respiratory delivery of another glycopeptide (Lactomorphin) [24]. Thus, we choose Trehalose again for formulating Ang (1—7) and PNA5 in their co-SD counterparts. The absence of internal hydrogen bonds in Trehalose allows a flexible formation of hydrogen bonds with drugs in general. This study shows that the presence of Trehalose in the solution has enabled Ang (1—7) and PNA5 molecules to be encapsulated into small, smooth spherical particles at a low pump rate. Simultaneously, pure compounds formed dimple or shriveled particles at this pumping rate; this is likely due to the hydrogen bonding between the Trehalose and these two compounds. Specifically, in the co-SD PNA5: Trehalose system, and that is due to the existence of the hydroxyl groups of the carbohydrate moiety in the PNA5, which may be allowed more bonding between Trehalose and PNA5; this assumption needs further investigation using other techniques such as nuclear magnetic resonance (NMR). Moreover, Trehalose seems to play a role in preventing the cracks from forming on the droplets’ surface during the evaporation process. These cracks would occur due to increased solvent vapor pressure during the spray drying process leading to dimpled or shriveled particle formation, as seen in the SD Ang (1—7) and SD PNA5 powders. Moreover, Trehalose is superior as an excipient for several reasons; stabilization against thermal stress [58], reduced hygroscopicity; and a high glass transition temperature [55]. Moreover, it is similar in structure to lactose monohydrate (the pulmonary sugar carrier) with a low rate of metabolism by bacteria due to its non-reducing 1,1′ linkage [55,58,59].

Furthermore, the feed (pump) rate generally impacts particle morphology, size, and the final product’s water content. In this study, we used a low feed rate (25%) in the experiments, which shown to be the optimum pump rate in terms of producing powders with smaller particle size and lower water content [24]; as it refers to transferring the feed solution into the nozzle per time. Thus, decreasing the pump rate results in smaller particle sizes as less fluid is provided. Therefore, the quantity of energy given by the air pressure and the temperature per droplet is high and more efficient in producing particles with lower residual water content due to sufficient drying [60].

Another factor that affected the powders’ particle size and water content is the feeding solvent; as mentioned above, using organic solvents (methanol specifically in this study) contributed to the production of smaller primary droplet sizes because of the lower surface tension (~22.5 mN/m). Residual water can significantly influence dry powder’s dispersion during aerosolization [28,29,46,55,61]. In this study, using an organic solvent as a feed solvent lowered the water content successfully in the solid-state as quantified by KF coulometric titration compared to the water content of raw counterparts’ powder. All SD and co-SD powders had a residual water content of ≤3.15% *w*/*w*; these residual water content values are considered suitable for inhalation dry powder formulation and necessary for effective particle delivery.

As the goal of this study was to produce dry powders of Ang (1—7) and PNA5 with favorable properties that enable them to target the respiratory tract (upper and lower), we started by testing the possibility of targeting the lower respiratory tract via aerosol dispersion performance using the NGI^®^ and the Neohaler™ DPI device. Excellent aerosol performance with optimal parameters was demonstrated. The results indicated that the formulated particles would be optimal for efficient and predominant deposition into the deep lung region for targeted delivery as inhaled nanoparticles/microparticles aerosolized powders. The lung deposition relates to particle aerodynamic behavior; the optimal MMAD for a particle is approximately 1–5 μm. In this study, the MMAD values for all formulations were less than 2.00 µm, which is considered an optimal value for targeting the smaller airways [61,62]. Furthermore, the FPF and RF values for all SD and co-SD powders were higher than their raw counterparts. Thus, there is a great potential the processed powders would be able to target the deep lung with high local concentration as they have relatively high ED% values in a range of (64–86%) besides all the optimal performance parameters mentioned above.

The other part of this study aimed to produce Ang (1—7) and PNA5 dry powders with properties suitable to target the brain through the olfactory route. Therefore, it was crucial to look carefully at the factors that affect nose-to-brain delivery and play essential roles in the particle deposition in the human nose. From an anatomic perspective, the olfactory epithelium is located at the top of the nasal cavity between the superior turbinate and the cribriform plate of the ethmoid bone [63]. Thus, a small fraction of therapeutics deposit in the olfactory region. From a formulation and delivery process perspective: particle size, airflow rate, and particle shape are significant factors that affect particle deposition. Many studies tested the particles deposition in the nose up to the olfactory generally and in the olfactory specifically. For example, it was reported in [64], 1 nm diffusive particles, and 10 μm inertial particles, are very easily trapped by the nasal vestibule due to dominant Brownian motion and inertial force, respectively. In comparison, a low olfactory deposition was observed for particles in the range of 10 nm to 2 μm [64].

On the other hand, it was found that particles of 100 nm penetrated the olfactory bulb and could be found in the brain, while particles of 900 nm did not penetrate the brain [65]. Another study [66] reported that the relationship between particle size (1–100 nm) and particle diffusivity, olfactory deposition efficiency was highest for the smallest particles in both humans and rats. Among the particles that do deposit, the larger nanoparticles have a higher probability of depositing in the olfactory region [66]. We can see a particle size cutoff could be essential for the delivery beyond the olfactory bulb from these studies. Although the particle size of processed Ang (1—7) and PNA5 powders in this study fall within a size range less likely to reach the olfactory region according to some research above, there is no specific particle size suitable for sure for olfactory depositions. Moreover, these two compounds are known to be potent; thus, even if a small amount of these compounds successfully reaches the olfactory region, hence, penetrate the brain through the Cerebrospinal fluid (CSF), it could cause the required treatment. All of these are possibilities, required to be tested in vivo in the coming future as our next step in this project. However, the properties of both the Ang (1—7) and PNA5 formulated particles (especially the co-SD binary systems with Trehalose) are more promising. A higher probability of these processed powders reaching the olfactory region due to the improved particle shape, smaller size and size distribution, and smoother surface morphology, as their raw counterparts are chunk-like powders with high potential to be trapped in the nose before reaching the olfactory.

There was an absence of crystallinity in raw Ang (1—7), raw PNA5, SD, and co-SD particles as reflected in the XRPD. DSC confirmed that the novel advanced inhalable microparticles and nanoparticles exhibited an apparent T_g_. Thus, the formation of the amorphous glassy state was also confirmed by visualizing the absence of birefringence through HSM. The DSC analysis shows high T_g_ and T_m_ values for all powders, which indicates the stability of these powder formulations at room and physiological temperatures. ATR-FTIR analysis and confocal Raman microscopy were performed for all powders before and after formulation to show the chemical composition and functional groups’ characteristics for each compound. Furthermore, we confirmed no change in the functional groups before and after the spray drying process for both compounds. Moreover, the co-SD particles’ analysis in their dry state using these two complementary analytical techniques confirmed the presence of the signature peaks of Ang (1—7) and trehalose in the co-SD Ang (1—7): Trehalose, and the presence of PNA5 and Trehalose characteristic peaks in their co-SD particles. Furthermore, Raman microscopy confirmed the particles’ chemical homogeneity by checking each powder’s spectra at three different spots.

To assess the safety of Ang (1—7) and PNA5, an in vitro cell viability resazurin assay was performed on human nasal septum (RPMI2650) cells, normal human astrocytes (NHA) cells, human blood–brain barrier hCMEC/D3 cells, H441 human lung airway cells, and A549 human alveolar epithelial cells after being exposed to different concentrations of Ang (1—7) and PNA5. The results show that Ang (1—7) and PNA5 are safe (non-toxic) up to 1000 μg/mL on nasal epithelium cells, endothelial brain, astrocyte brain, and epithelium lung cells. The safety of these two compounds in the tested cell lines is unsurprising results, as they are peptides known for their unique properties such as low toxicity. The same results were observed in the MucilAir cells, which represent a 3D model of the human airways. Furthermore, a degradation analysis of PNA5 [22] showed that this compound’s primary metabolites are the initial cleavage of the glucose followed by subsequent degradation at the Ang-(1-6) six peptide cleavage sites. Thus, this study showed no evidence that the modification via glycosylation yielded harmful degradation products or products that are not produced by the natural degradation of endogenous Ang (1—7). Thus, the expected molecules upon degradation in vivo would be mainly the amino acids and the sugar moiety, which are safe; this is in good agreement with our cell viability results. The in vitro 2D and the 3D TEER results showed that cells recovered within hours after the exposure of the formulations.

This study shows the potential of these formulated Ang (1—7) and PNA5 powders to target the respiratory tract; this would help treat many lung diseases besides causing the antinociceptive effect. The relationship between the RAS pathway and COVID-19 is not yet completely clear; however, the expression of the Mas receptor in the central nervous system and lungs and delivering these two compounds, which are agonists to this receptor locally as DPI, can be a potential therapeutic approach to treat the COVID-19 complications.

## 5. Conclusions

This comprehensive and systematic study reports for the first time on rationally designed SD Ang (1—7), SD PNA5, co-SD Ang (1—7):Trehalose (25:75), co-SD PNA5:Trehalose (25:75) microparticulate/nanoparticulate for targeted respiratory delivery. Ang (1—7) and PNA5 were synthesized successfully with high purity (≥98%), then formulated via particle engineering using advanced spray drying approaches. Comprehensive physicochemical characterization illustrated an amorphous glassy state retained following organic solution advanced spray drying, a clear and high glass transition, and melting temperatures. Moreover, the formulated particles exhibited a spherical particle shape, relatively smooth particle surface, and very low residual water content that minimized inter-particulate interactions, and enhanced aerosol dispersion efficiency as DPIs; hence, less molecular mobility, low reactivity increases physical and chemical stability. The safety (non-toxicity) and biocompatibility of Ang (1—7) and PNA5 were confirmed by in vitro 2D/3D cell viability and 2D/3D TEER at the ALI using different human cell lines.

## Figures and Tables

**Table 1:** Spray drying conditions for organic solution advanced spray drying in closed-mode under ultra-high purity nitrogen gas for (Ang (1—7), PNA5, Trehalose) spray-dried systems, and (co-SD Ang (1—7):Trehalose, PNA5:Trehalose) co-spray-dried systems.

Formulations (Powder Composition = Molar Ratio)	SD Ang (1—7) SD PNA5 co-SD Ang (1—7):Trehalose (25:75) co-SD PNA5:Trehalose (25:75)	co-SD PNA5:Trehalose (20:80)
Feed concentration in solvent (% *w*/*v*)	0.125	2.5
Solvent	Methanol	Methanol
Aspiration gas: ultra-high purity nitrogen gas (L/hour)	670 (55 mm in height)	670 (55 mm in height)
Inlet temperature (°C)	150	150
Outlet temperature (°C)	47–53	60
Inert loop temperature (°C)	−15	−15
Pump rate (%)	Low (25%) (7.5 mL/min)	Low (25%) (7.5 mL/min) and Medium (50%) (15 mL/min)
Aspirator (%)	100 (35 m^3^/h)	100 (35 m^3^/h)

**Table 2:** Solubility of Ang (1—7) and PNA5 in different solvents (mean ± standard deviation, *n* = 3) by experimental and computational methods.

Solvent	Solubility of Ang (1—7) mg/mL ± Std dev	Ang (1—7) LogS *	Ang (1—7) LogS Calculated by MOE	Ang (1—7) Solubility Category According to USP Definition	Solubility of PNA5 mg/mL ± Std dev	PNA5 LogS *	PNA5 LogS Calculated by MOE	PNA5 Solubility Category According to the USP Definition
H_2_O	174.03 ± 0.19	−0.71	−0.82	Freely soluble (FS)	275.33 ± 2.31	−0.58	−0.53	Freely soluble (FS)
PBS	165.38 ± 0.34	−0.73	-	Freely Soluble (FS)	305.05 ± 0.22	−0.54	-	Freely soluble (FS)
NS	140.37 ± 0.32	−0.81	-	Freely soluble (FS)	269.33 ± 1.53	−0.59	-	Freely soluble (FS)
Methanol	1.00 ± 0.005	−2.95	-	Slightly soluble (SS)	0.34 ± 0.01	−3.48	-	Very slightly soluble (VSS)
Ethanol	0.160 ± 0.001	−3.75	-	Very slightly soluble (VSS)	0.12 ± 0.003	−3.94	-	Very slightly soluble (VSS)

LogS: Log Solubility. * The experimental solubility (S) in mMole unit.

**Table 3:** Particle sizing using image analysis on SEM micrographs (*n* ≥ 100 particles).

Powder Composition (Molar Ratio)	Mean Size (μm)	Size Range (μm)
Raw Ang (1—7)	unmeasurable	-
Raw PNA5	unmeasurable	-
SD-Ang (1—7)	0.86 ± 0.42	0.31–2.62
SD-PNA5	0.78 ± 0.42	0.24–2.48
Co-SD Ang (1—7):Trehalose (25:75)	0.56 ± 0.21	0.20–1.25
Co-SD PNA5:Trehalose (25:75)	0.59 ± 0.35	0.10–1.72

**Table 4:** DSC thermal analysis (*n* = 3, mean ± standard deviation).

Parameter	Raw Ang (1—7)	SD Ang (1—7)	Raw PNA5	SD PNA5	Raw Trehalose	SD Trehalose	co-SD Ang (1—7):Trehalose (25:75)	co-SD PNA5:Trehalose (25:75)
T_g_ onset (°C)	165.67 ± 4.43	170.16 ± 2.54	162.3 ± 3.47	168.37 ± 1.96	-	37.53 ± 1.43	143.12 ± 2.21	155.19 ± 2.60
T_g_ mid (°C)	169.13 ± 6.54	175.10 ± 2.90	167.45 ± 3.66	172.78 ± 1.33	-	41.45 ± 0.70	143.21 ± 2.13	155.26 ± 2.61
T_g_ End (°C)	173.99 ± 0.09	180.1 ± 2.28	170.05 ± 3.85	174.45 ± 2.41	-	45.68 ± 1.63	143.28 ± 1.96	155.32 ± 2.54
∆C_p_ (J/g.)	1.80 ± 0.40	1.57 ± 0.04	1.19 ± 0.10	1.47 ± 0.00	-	0.59 ± 0.05	0.70 ± 0.12	1.26 ± 0.10
Endotherm1 onset (°C)	217.54 ± 11.06	219.81 ± 3.92	231.98 ± 7.43	220.52 ± 4.61	94.70 ± 0.08	145.81 ± 2.92	178.41 ± 2.35	169.42 ± 1.42
Endotherm1 peak (°C)	218.45 ± 10.84	221.29 ± 3.93	232.45 ± 7.21	220.70 ± 4.47	95.85 ± 0.12	146.3 ± 2.57	185.82 ± 1.65	179.53 ± 0.86
∆H_1_(Enthalpy) J/g	45.96 ± 5.34	41.05 ± 2.67	81.37 ± 20.28	52.54 ± 4.96	91.92 ± 7.79	3.39 ± 0.68	27.08 ± 1.96	8.88 ± 0.66
Endotherm2 onset (°C)	-	-	-		141.32 ± 1.20	182.90 ± 4.60	209.65 ± 8.03	204.26 ± 7.15
Endotherm2 peak (°C)	-	-	-		141.01 ± 1.14	189.76 ± 3.88	213.28 ± 7.12	207.65 ± 8.25
∆H_2_ (Enthalpy) J/g	-	-	-		106.90 ± 6.15	20.66 ± 2.30	34.02 ± 9.16	58.76 ± 28.99
Endotherm3 onset (°C)	-	-	-		196.54 ± 1.10	-	-	-
Endotherm3 peak (°C)	-	-	-		204.24 ± 1.10	-	-	-
∆H_3_ (Enthalpy) J/g	-	-	-		118.33 ± 4.17	-	-	-
Exotherm (T_C_) °C	-	-	-		-	99.41 ± 0.82	114.85 ± 8.68	142.71 ± 1.04

**Table 5:** Water content of SD Ang (1—7), SD PNA5, SD Trehalose, co-SD Ang (1—7): Trehalose, co-SD PNA5: Trehalose microparticulate/nanoparticulate powder systems and their raw counterparts analyzed via Karl Fisher coulometric titration (mean ± standard deviation, *n* = 3).

System	Water % (*w*/*w*)
Raw Ang (1—7)	4.03 ± 0.13
Raw PNA5	4.64 ± 0.18
Raw Trehalose	8.55 ± 1.50
SD Trehalose	4.18 ± 0.54
SD Ang (1—7)	3.15 ± 0.13
SD PNA5	2.27 ± 0.13
Co-SD Ang (1-—7):Trehalose (25:75)	2.13 ± 0.44
Co-SD PNA5:Trehalose (25:75)	2.99 ± 0.92

**Table 6:** In vitro aerosol dispersion performance as DPIs (*n* = 3, mean ± standard deviation).

Powder Formulation Composition (Molar Ratio)	Emitted Dose (ED) (%)	Fine Particle Fraction (FPF) (%)	Respirable Fraction (RF) (%)	MMAD (μm)	GSD
Raw Ang (1—7)	100.74 ± 2.69	7.75 ± 0.44	60.75 ± 0.48	5.53 ± 0.91	7.06 ± 0.75
Raw PNA5	101.52 ± 5.91	3.33 ± 0.15	41.26 ± 0.76	9.01 ± 0.64	2.89 ± 0.21
SD Ang (1—7)	79.80 ± 2.06	44.45 ± 3.85	97.35 ± 2.00	1.72 ± 0.03	1.76 ± 0.07
SD PNA5	64.45 ± 1.73	50.52 ± 8.18	96.93 ± 2.39	1.99 ± 0.06	1.97 ± 0.17
Co-SD Ang (1—7): Trehalose 25:75	83.70 ± 1.53	50.21 ± 1.58	95.71 ± 1.57	1.70 ± 0.08	1.81 ± 0.10
Co-SD PNA5: Trehalose 25:75	86.26 ± 0.22	32.49 ± 0.28	91.26 ± 0.31	2.09 ± 0.02	1.81 ± 0.03

## Data Availability

The data presented in this study are available on request from the Corresponding Author.
